# Mechanically Activated Luminescence in Polyurethanes Incorporating Calixarene Mechanophores

**DOI:** 10.1002/anie.9927716

**Published:** 2026-04-27

**Authors:** Lucia Visieri, Alessandro Casnati, Laura Baldini, José Augusto Berrocal

**Affiliations:** ^1^ Institute of Chemical Research of Catalonia (ICIQ) The Barcelona Institute of Science and Technology (BIST) Tarragona Spain; ^2^ Department of Chemistry Life Sciences and Environmental Sustainability University of Parma Parma Italy; ^3^ Catalan Institution for Research and Advanced Studies (ICREA) Barcelona Spain

**Keywords:** calixarenes, mechanochromic polyurethanes, mechanoluminescence, mechanoresponsive polymers, supramolecular mechanophores

## Abstract

The conformational properties of *cone* calix[4]arenes are exploited to develop the first example of a calixarene‐based mechanoluminophore. By functionalizing the distal positions at the upper rim of a calix[4]arene with pyrene moieties, we produce a macrocycle incorporating two 1,6‐bis(phenylethynyl)pyrene fluorophores. The flexibility of the calix[4]arene scaffold facilitates the formation of intramolecular ground‐state excimers involving the pyrene units, as evidenced by ^1^H NMR and fluorescence spectroscopy. Incorporating the calixarene mechanoluminophore into linear polyurethanes yields uniform films that exhibit the characteristic pyrene excimer emission. When these films are stretched, either manually or using a universal tensile tester, a fluorescence shift from green–yellow to blue is observed under irradiation with UV light. Such fluorescence change is reversible and repeatable over numerous stress and release cycles. We attribute this behavior to conformational changes in the calix[4]arene triggered by mechanical force, and in particular to the temporary dissociation of the intramolecular pyrene excimers into pyrene monomers. Thus, the flexible calix[4]arene scaffold enables the reversible separation of the two fluorophores while preserving their spatial proximity. We propose that this design concept can provide general guidelines for creating supramolecular mechanophores free of intermolecular aggregation effects.

## Graphical abstract

The conformational flexibility of a calix[4]arene is exploited to obtain a reversible mechanoluminophore featuring a pyrene excimer couple at the upper rim. Stretching a linear polyurethane incorporating such mechanophore results in luminescence color change from yellow–green to blue, consistent with a force‐induced conformational motion of the calixarene scaffold that dissociates the excimer into two pyrene units.

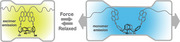

## 1 Introduction

**FIGURE 1:**
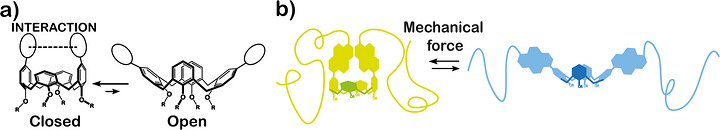
(a) Conformational equilibrium between the two *pinched cone* conformations of calix[4]arenes. The presence of attractive interactions between distal groups at the upper rim favors the closed structure. (b) Schematic representation of a polyurethane chain incorporating a calixarene‐based mechanophore. Mechanical stress induces the dissociation of the pyrene dimer at the upper rim of the calixarene, resulting in a change from the excimer emission (yellow‐green) to the monomer emission (blue).

Although they were first reported in the 1940s [1], calixarenes gained prominence only in the 1970s and 1980s and have since then become crucial scaffolds in supramolecular chemistry [2]. Their versatile framework has facilitated the design of receptors for ion transport [3], catalysis [4, 5, 6], and recognition of biomacromolecules [7], among other applications. A key factor in their success is their inherent conformational flexibility, which can be modulated—or even locked—through tailored functionalization, allowing access to preorganized molecular architectures. For example, calix[4]arenes can be maintained in a *cone* conformation by proper lower rim alkylation with groups bulkier than ethyl moieties. This *cone* conformation is fixed, yet retains a degree of conformational freedom that causes a rapid interconversion between two equally stable *pinched cone* forms [8]. Strategically placing judiciously chosen substituents at the upper rim can bias this equilibrium toward a single conformer via hydrogen bonding, π–π, or electrostatic attractive interactions (Figure 1a) [9]. This inherent flexibility has been explored by covalently linking distal positions at the upper rim with one‐ to three‐atom bridges [10, 11, 12, 13, 14], as well as through intramolecular reactivity, such as the Cannizzaro reaction (i.e., aldehyde disproportionation) of formylated calixarenes [15].

The calix[4]arene conformational equilibrium can also be influenced by external stimuli. For example, pyrene‐ and amine‐functionalized *cone* calix[4]arenes, where pyrenes and amino groups are alternately positioned on the upper rim, exhibit protonation‐controlled switching between closed and open forms, each with distinct emission properties [16]. In another example, fluorophore‐containing calix[4]arenes displayed thermoresponsive modulation of fluorescence [17]. Collectively, these examples highlight the stimulus‐responsive properties of calix[4]arenes, while also pointing out that their behavior under mechanical force remains largely unexplored. To date, only one study has reported a similar vase‐kite conformational switch in a cavitand‐crosslinked polyurethane under tensile stress; however, the response associated with the conformational change was subtle and visually challenging to detect [18]. We therefore sought to design a system that would produce a clear, optically visible signal associated with a force‐triggered conformational change.

Luminescence serves as a powerful tool for monitoring the force‐triggered disruption of interactions between dyes. Early strategies relied on blending excimer‐forming dyes into semi‐crystalline polymers [19], where stretching disrupted dye aggregates and enhanced monomer emission. These systems were effective, but offered limited molecular‐level control as the dyes were physically dispersed in the polymer matrix. Consequently, the optical response depended strongly on the dye concentration and its spatial distribution within the polymer, both of which were sensitive to polymer morphology and processing strategy. Subsequent approaches introduced excimer‐forming dyes covalently incorporated into the polymer backbone and constrained by linkers that hold chromophores in close proximity [20]. This design promotes excimer formation and prevents irreversible separation upon application of force, thus enabling reversible mechanochromic behavior. However, avoiding intermolecular aggregation within dyes remained challenging. To overcome these challenges, highly luminescent dyes have been incorporated into macrocyclic structures, such as cyclophanes [21], paracyclophanes [22], and rotaxanes [23]. These architectures pre‐organize the fluorophores in close spatial proximity, allowing exclusively intramolecular and reversible excimer association–dissociation. However, they typically require considerable synthetic effort. In this regard, calixarenes offer an attractive alternative for the construction of luminescent mechanophores: they are synthetically accessible, conformationally adaptable, and readily functionalized. Their preorganized cavities provide a natural platform for installing excimer‐forming dyes, paving the way for coupling mechanical stimuli with a direct, visible luminescent response.

Building on this rationale, we hypothesized that embedding calixarene‐based mechanophores into polymer matrices would enable mechanical control over their conformational equilibrium, translating macroscopic force into an optical signal.

## 2 Results and Discussion

### 2.1 Mechanophore Design and Synthesis

As previously mentioned, a key characteristic of the calix[4]arene skeleton we aimed to design is its pinched *cone* conformation (Figure 1a). The latter can be achieved through exhaustive lower rim alkylation with propyl moieties. Our design would then be completed by distal pyrene units at the upper rim of the macrocycle, which should further favor the *closed* *pinched cone* conformation by forming a ground‐state pyrene excimer (Figure 1b).

**FIGURE 2:**
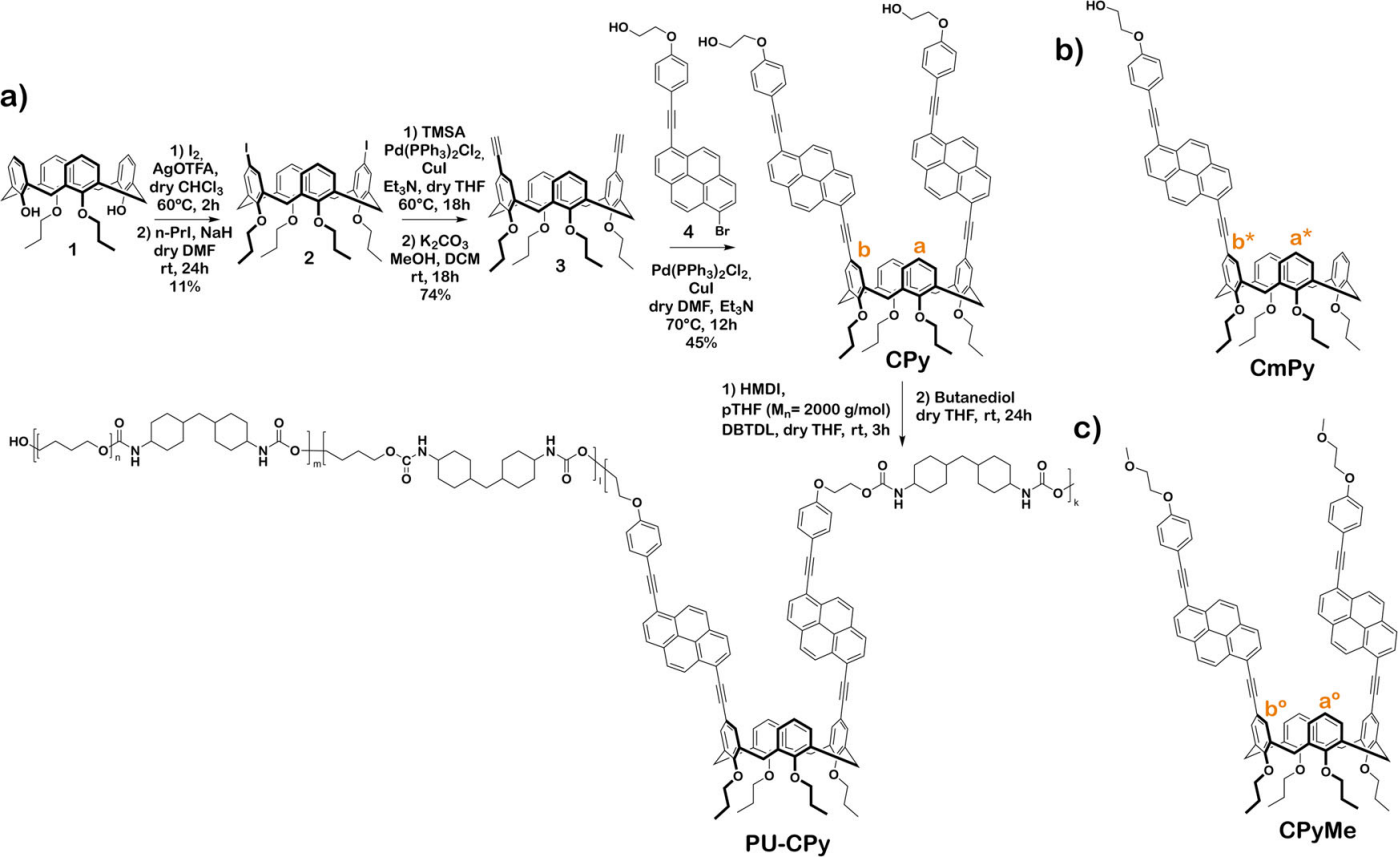
(a) Synthesis of **CPy** and **PU‐CPy**. The molecular structure of **PU‐CPy** shown in the figure is a representative **CPy**‐containing structure among the multiple statistical products formed during synthesis and differing by the number and sequence of monomers. Molecular structures of (b) **CmPy** and (c) **CPyMe**. Labels a, b, a*, b*, a°, and b° refer to aromatic protons discussed in Figure 3.

We translated these features into the design of **CPy** (Figure 2a). The latter was synthesized in three steps starting from dipropoxycalix[4]arene **1** [8] (Figure 2a), which was treated first with iodine and silver trifluoroacetate, and then with *n*‐iodopropane under strong basic conditions to obtain **2**, featuring two iodine groups in distal positions at the upper rim and exhaustive alkylation at the lower rim. A first Sonogashira coupling between **2** and trimethylsilyl acetylene (TMSA), followed by deprotection of the trimethylsilyl groups, afforded diethynyl derivative **3** in good yields. The rigidity of the alkyne linker was intended to minimize residual mobility that could interfere with mechanical force transduction. Compound **3** was then subjected to a second Sonogashira coupling with pyrene derivative **4** (synthesis described in the SI, pages S6 and S7) to afford **CPy**, comprising 2‐hydroxyethoxy moieties to favor its covalent incorporation into polyurethanes (vide infra).

Following similar synthetic routes, we also prepared control compounds **CPyMe** (Figure 2c), having the hydroxyl groups protected as methyl ethers, and **CmPy** (Figure 2b), featuring only one pyrene unit. The syntheses of **CPyMe** and **CmPy** are documented in the SI (pages S8–S11). All newly isolated compounds were characterized by proton and carbon nuclear magnetic resonance (^1^H and ^13^C NMR) spectroscopy, and mass (MS) spectrometry (SI, pages S29–39).

**CPy** was then covalently incorporated into linear polyurethane elastomer **PU‐CPy** via a polyaddition reaction. First, a prepolymer was formed by reacting 4,4′‐methylene‐bis(cyclohexyl isocyanate) (HMDI), a telechelic high molecular weight (*M_n_* ≈ 2000 g·mol^−^^1^) poly(tetrahydrofuran)‐diol (pTHF), and **CPy** (Figure 2a). 1,4‐butanediol (BDO) was then used as a chain extender. To minimize potential intermolecular interactions in the solid state, the **CPy** content was kept low at 0.023 wt% (corresponding to 2.07 mol% and 0.022 **CPy** units per polymer chain). The resulting polymer was precipitated in hexane, collected by filtration, and subsequently washed with hexane to remove any unbound luminophore. Size exclusion chromatography (SEC) analysis revealed a number‐average molar mass (*M_n_*) of 133 kDa and a dispersity (*Đ*) of 1.9 for **PU‐CPy**, in line with a step‐growth polymerization. A control polymer **PU‐CmPy** (*M_n_* = 173 kDa, *Đ* = 1.7), comprising equal amounts of HDMI, pTHF, and BDO, and 0.033 wt% of **CmPy** (equal to 5.74 mol% or 0.056 **CmPy** units per polymer chain) to maintain a comparable concentration of pyrene units, was also prepared (page S11 and S12). Control polyurethane (**PU**) (*M_n_* = 58 kDa, *Đ* = 1.8) samples devoid of any luminescent molecule (i.e., composed solely of HDMI, pTHF, and BDO) were also synthesized (pages S12). Finally, the pristine matrix **PU** was blended with concentrations of **CPyMe** equal to 0.025 wt% (**0.025CPyMeinPU**), 0.05 wt% (**0.05CPyMeinPU**), and 0.1 wt% (**0.1CPyMeinPU**) (page S12).

Uniform films of all polymer samples were prepared by compression molding at 120°C and 4 tons for 5 min, resulting in films with thicknesses of approximately 220–230 µm. Differential scanning calorimetry (DSC) revealed a glass transition temperature (*T_g_*) of −43–−44°C for all samples, with no evidence of melting/crystallization events in the heating/cooling runs (i.e., lack of crystalline phases) (Figures S1–S3). All materials displayed thermal stability up to 300–330°C, as shown by thermogravimetric analysis (TGA) (Figures S4–S6). Under tensile loading (Figures S7–S9), the samples withstood strain up to 500% (strain rate 2.5% s^−1^), reached maximum stresses of 3–6 MPa, and showed Young's moduli within the range 3.9–7.2 MPa. A summary of all polyurethane formulations and more relevant characteristics is shown in Table 1. The comparable thermal and mechanical properties of **PU‐CPy**, **PU‐CmPy**, and **0.025–0.1CPyMeinPU** suggest that covalent incorporation of the luminophores does not significantly alter the bulk physical properties of the polyurethane.

**TABLE 1:** The determined molar mass (*M_n_*), dispersity (*Đ*), weight (wt%) and molar (mol%) percentages of calix[4]arene‐derivative loading, along with the average number of calix[4]arene‐derivative per polymer chain (u. per chain), glass transition temperature (*T_g_*), and Young's modulus for each polyurethane formulation.

Formulation	*M_n_* (kDa)	*Đ*	wt%	mol%	u. per chain	*T_g_* (°C)	Young's modulus (MPa)
**PU‐CPy**	133	1.9	0.023	2.07	0.022	−44	3.96
**PU‐CmPy**	173	1.7	0.033	5.74	0.056	−43	4.56
**0.025CPyMeinPU**	58	1.8	0.025	1.04	—	−44	7.22
**0.05CPyMeinPU**	58	1.8	0.05	2.09	—	−44	7.22
**0.1CPyMeinPU**	58	1.8	0.1	4.17	—	−44	7.22

### 2.2 Conformational and Photophysical Properties of CPy

**FIGURE 3:**
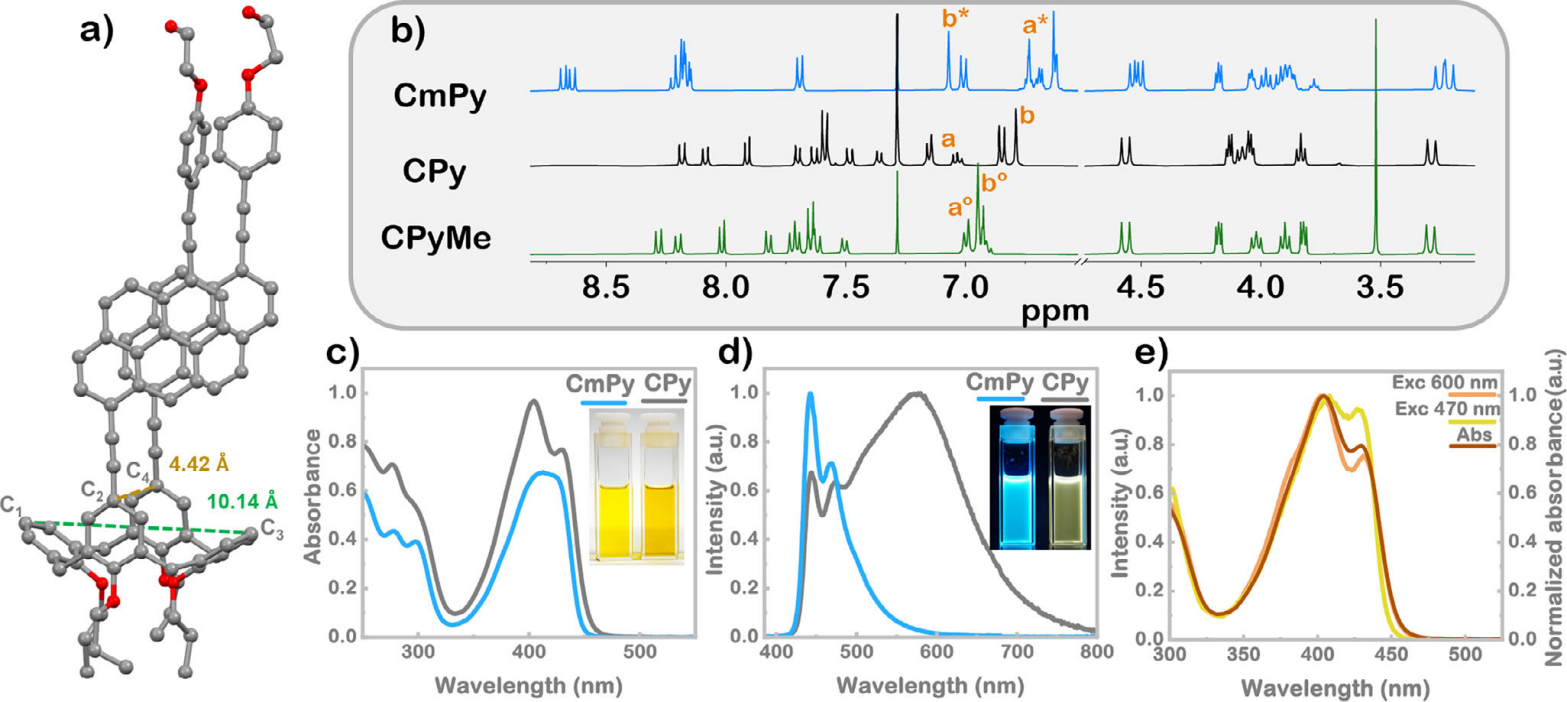
(a) Solid‐state structure of **CPy** (hydrogen atoms and two DCM molecules were omitted for clarity). (b) Comparison between selected regions of the ^1^H NMR spectra of **CmPy**, **CPy**, and **CPyMe** recorded in CDCl_3_ at 400 MHz and 25°C. Signals assignment, indicated by letters, refers to Figure 2. (c) Absorption spectra of 10^−5^ M CH_2_Cl_2_ solutions of **CPy** and **CmPy** and photos of the analyzed cuvettes. (d) Emission spectra of 10^−6^ M CH_2_Cl_2_ solutions of **CPy** and **CmPy** under irradiation with 380 nm light, and photos of the analyzed cuvettes under irradiation with 365 nm light. (e) Comparison between fluorescence excitation spectra of a 10^−6^ M CH_2_Cl_2_ solution of **CPy** at 600 and 470 nm, and absorption spectrum of a 10^−5^ M CH_2_Cl_2_ solution of **CPy**.

X‐ray quality crystals of **CPy** (yellow lamellae) were grown by solvent layering, using CH_2_Cl_2_ as the solvent and hexane as the antisolvent. X‐ray diffraction (XRD) results shown in Figure 3a evidence the closed *pinched cone* conformation adopted by **CPy** in the solid‐state. Indeed, the distance between *C1* and *C3* (10.14 Å) is more than twice that between *C2* and *C4* (4.42 Å). The two pyrene units appear to be in close proximity and disposed in a stacked arrangement similar to AB‐stacked bilayer graphene [24]. The average distance between eclipsed C atoms of the two pyrene rings is 3.48 Å. The interplanar separation between the pyrene units is slightly shorter in the region of the dimer closest to the calixarene scaffold, whereas it increases on the opposite side due to a T‐shaped π−π interaction between the terminal 2‐hydroxyethoxy phenol rings (Figure S10). The OH groups of the latter units are involved in H‐bond interactions with the corresponding units of neighboring molecules in the crystal lattice.

Next, we investigated by ^1^H NMR spectroscopy whether the close proximity between the two pyrenes of **CPy** observed in the solid state could also occur in solution. The ^1^H NMR spectrum of a 5 mM solution of **CPy** was compared with those of equally concentrated **CmPy** and **CPyMe** solutions (selected regions shown in Figure 3b; full spectra comparison shown in Figure S11).

The pyrene protons of **CPy** appear as eight non‐equivalent doublets (7.3–8.2 ppm) at room temperature (black spectrum in Figure 3b). The protons of the substituted aromatic rings of the calixarene resonate at higher fields (6.8 ppm; signal “b” assigned in Figure 2a) compared to those of the non‐substituted rings (7.1 ppm; signals “a” assigned in Figure 2a), a diagnostic signature of the *closed pinched cone* conformation [9].

A comparison between **CPy** and the single‐pyrene reference compound **CmPy** shows that the pyrene signals of **CPy** are upfield shifted with respect to **CmPy** (8.1–8.7 ppm, blue line in Figure 3b). This shift likely arises from the magnetic anisotropy exerted by one pyrene on the other in the π–π stacked arrangement [25]. DOSY spectra of **CPy** and **CmPy** (Figures S12 and S13) rule out intermolecular aggregation at the working concentration (5 mM). Finally, the comparison between **CPy** and **CPyMe** (green line in Figure 3b) reveals that when the terminal hydroxy groups are replaced by methoxy moieties (as in **CPyMe**), the pyrene units remain in close proximity. However, their resonances show an average downfield shift of 0.12 ppm relative to what was observed with **CPy**, suggesting a modest increase in the average distance between the pyrene units. Overall, these results support that **CPy** resides in a *closed* *pinched cone* conformation also in solution. The hydrogen bonds are not essential for holding the *closed* *pinched cone* conformation, although they provide further contribution to stabilizing it.

The optical properties of **CPy** and **CmPy** were analyzed by UV–visible (Figure 3c) and fluorescence (Figure 3d) spectroscopies in dilute dichloromethane solutions (10^−5^ M and 10^−6^ M, respectively). **CPy** has an absorption band with a maximum at 405 nm and a shoulder at 430 nm, while **CmPy** shows a broader absorption band with a maximum at 412 nm. The non‐superimposable absorption profiles of the two compounds suggest, once again, dimerization of the pyrenes at the upper rim of **CPy**. Even more pronounced differences appear in the emission spectra of the two molecules (Figure 3d). **CmPy** emits in a narrow region in the blue portion of the visible spectrum (400–600 nm), with a maximum at 442 nm. In contrast, **CPy** exhibits a broad (400–800 nm) and red‐shifted emission band with a maximum at 580 nm, which is characteristic of excimer emission [21, 22]. The excimer emission band is accompanied by two, less intense, local maxima at 444 nm and 475 nm that are reminiscent of the pyrene monomer emission observed for **CmPy**. The spectral features of **CPy** suggest the existence of a dynamic equilibrium between monomeric and dimeric pyrenes in solution—similar to the one shown in Figure 1b—with the equilibrium strongly shifted towards the ground‐state dimer. As the equilibrium is detectable only by UV–vis and fluorescence—the ^1^H NMR spectra did not show signals ascribable to such phenomenon—we speculate that the rate of the exchange process occurs on a fast timescale [26]. This interpretation is corroborated by fluorescence excitation spectroscopy (Figure 3e). When the emission is monitored at 600 nm, the excitation profile closely matches the absorption spectrum of **CPy**. However, the excitation profile diverges significantly from the absorption spectrum of **CPy** and becomes more reminiscent of the absorption spectrum of **CmPy** upon monitoring the emission at 470 nm. Moreover, the intensity of the excimer‐like emission of **CPyMe** is reduced but not entirely suppressed, confirming the pivotal role of the aromatic interaction between pyrene units in holding the calixarene in the *closed pinched cone* conformation (Figure S14).

Concentration‐dependent (1–8 µM) fluorescence studies of **CPy** solutions in CH_2_Cl_2_ showed no variation in the emission profile upon dilution, further ruling out possible intermolecular aggregation (Figure S15).

Finally, emission lifetimes measurements revealed a monoexponential decay with a lifetime of 1.3 ns for the emission of **CmPy** at 440 nm, which is typical of pyrene monomers (Figure S16) [21, 22, 27]. In contrast, the emission decay of **CPy** monitored at 600 nm is significantly longer and fits a biexponential model, with lifetimes of τ_1_ = 8.6 ns and τ_2_ = 34.5 ns, consistent with reported values for pyrene excimers [22, 27]. When monitored at 440 nm, the emission decay of **CPy** also fits a biexponential function, with τ_1_ = 1.3 ns and τ_2_ = 9.8 ns, further confirming the coexistence of both monomer and excimer species in solution (Figure S16).

To sum up, all the measurements discussed thus far strongly suggest (i) the occurrence of a rapid exchange between a dimeric and monomeric form of the two pyrenes at the upper rim of the calixarene of **CPy**, and (ii) the higher stability of the conformer that favors ground‐state interaction.

### 2.3 Mechanochromic Properties of the Polyurethane Formulations

**FIGURE 4:**
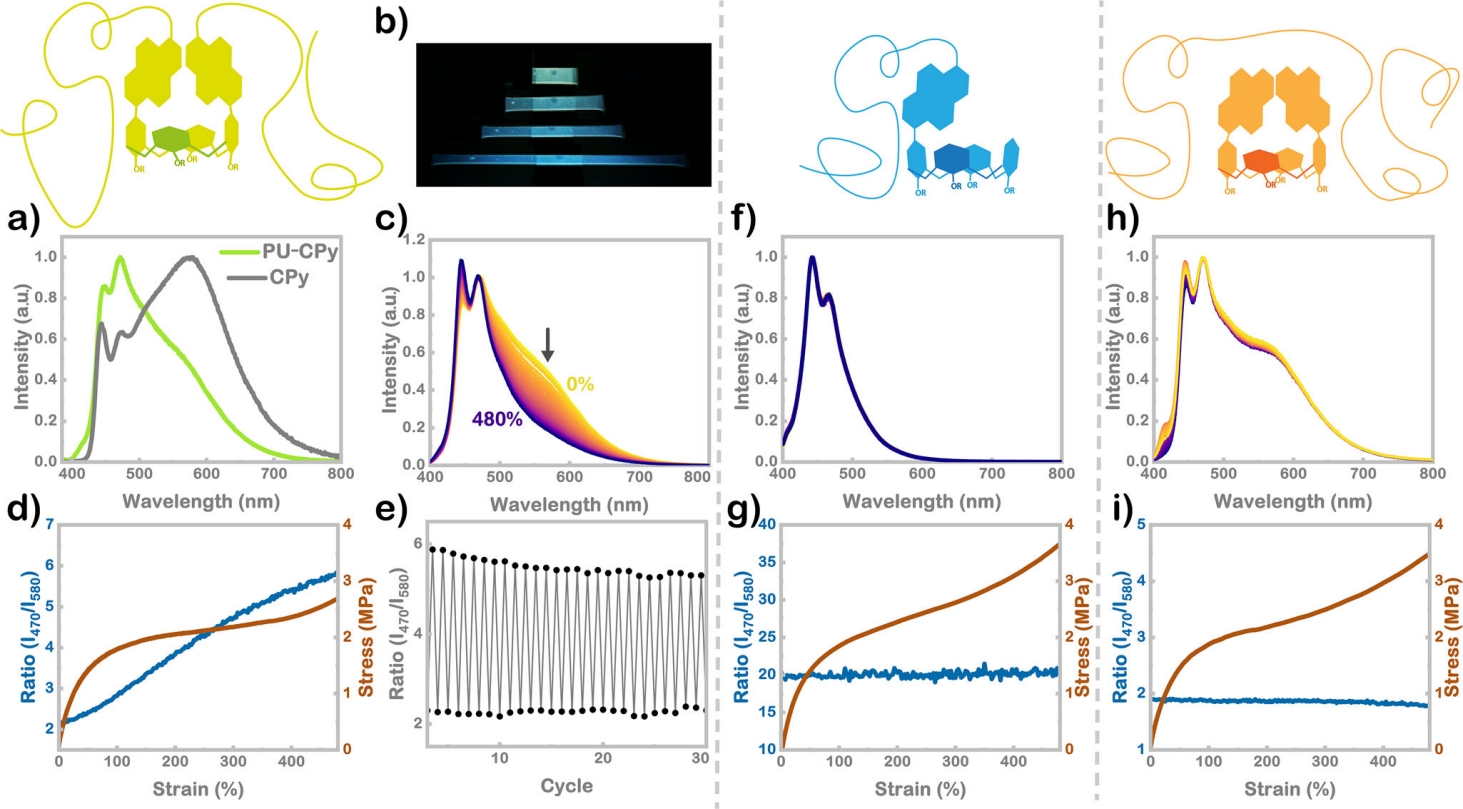
(a) Comparison between the emission profiles of **PU‐CPy** under 365 nm irradiation and 10^−6^ M **CPy** in DCM under 380 nm irradiation. (b) Photographs of the compression molded film (**PU‐CPy**) under UV light (365 nm) at strains ranging from 0% to 300%. (c) Emission spectra of a **PU‐CPy** film upon uniaxial tensile deformation from 0% (yellow profiles) to 480% (purple profiles) strain, under 365 nm irradiation, strain rate 2.5% s^−1^. (d) Correlation between stress–strain curves and 𝐼_470_/𝐼_580_ ratio in a **PU‐CPy** film. (e) Monomer‐to‐excimer ratio (𝐼_470_/𝐼_580_) in the stretched (480%) and relaxed (0%) states of **PU‐CPy** recorded over 30 cycles (strain rate 30% s^−1^), the first two cycles were omitted due to hysteresis. (f) Emission spectra of a **PU‐CmPy** film upon uniaxial tensile deformation from 0% (yellow profiles) to 480% (purple profiles) strain, under 365 nm irradiation, strain rate 2.5% s^−1^; (g) correlation between stress–strain curves and 𝐼_470_/𝐼_580_ ratio in a **PU‐CmPy** film. (h) Emission spectra of **CPyMeinPU0.025** films upon uniaxial tensile deformation from 0% (yellow profiles) to 480% (purple profiles) strain under 365 nm irradiation, strain rate 2.5% s^−1^; (i) correlation between stress‐strain curves and 𝐼_470_/𝐼_580_ ratio in a **CPyMeinPU0.025** film.

The incorporation of **CPy** into **PU‐CPy** was studied via photophysical characterization of **PU‐CPy** films and CH_2_Cl_2_ solutions (Figures 4a and S17, respectively). Both the emission profiles of **PU‐CPy** in solution and in the solid state are dominated by the two narrow bands with maxima at 443 and 470 nm, and exhibit an additional broad band around 580 nm, suggesting the coexistence of pyrene monomer and excimers, as previously observed with **CPy**. Nevertheless, the excimer emission band in the solid state is much less intense than in solution. We hypothesize that the steric hindrance imposed by the polymer chains reduces the dynamic motion of the luminophores in **PU‐CPy** and shifts the equilibrium of Figure 1b towards the monomeric state, albeit excimers are still present. The difference between the relative intensities of the vibronic bands of **CPy** and **PU‐CPy** film at 433 and 470 nm is attributed to inner filter effects. Indeed, an overlap between **CPy** absorption and emission spectra is present in the 420–460 nm range, which causes the radiation emitted within this range to be re‐absorbed before reaching the detector [28]. The phenomenon is much more intense for concentrated samples, and extremely common in solid‐state fluorescence measurements. Overall, the experiments confirmed the presence of **CPy** in the polymer backbone, although the photophysical properties of **CPy** were partially altered after incorporation into the polymer.

Next, we qualitatively investigated the emission properties of **PU‐CPy** films by visually monitoring their fluorescence under irradiation with 365 nm light (λ_ex_). Gratifyingly, the film exhibited distinct fluorescence color changes upon stretching, progressively shifting from yellow‐green in the stress‐free state to blue at higher strains (Figure 4b). Moreover, the strain‐dependent fluorescence changes appeared to be repeatable upon performing stretch‐release cycles (Supporting Movie S1).

These qualitative experiments were further confirmed by in situ fluorescence measurements carried out during uniaxial tensile deformation at a strain rate of 2.5% s^−1^ in which **PU‐CPy** films were pulled from 0% to 480% strain (Figure 4c). At low strains, the spectra display a prominent excimer emission band centered at 580 nm (yellow profiles), consistent with the previous discussion regarding the incorporation of **CPy** into **PU‐CPy** (cf. Figure 4a). However, the intensity of the excimer band diminishes and eventually disappears upon stretching to 480% strain, leaving only the monomeric contribution (λ_max_ = 443 and 470 nm) to the emission profile (purple lines). This behavior is consistent with the physical dissociation of the pyrene dyes triggered by the transduction of mechanical force from the polymer chains to the mechanophores. To quantify the mechanically‐induced activation of the **CPy** units in **PU‐CPy**, we employed a ratio‐metric approach [21, 29], resorting to the ratio of the intensities of the monomer and excimer emissions at 470 and 580 nm (𝐼_470_/𝐼_580_), respectively. This approach mitigates the influence of changes in the optical path length due to film thinning during deformation. Figure 4d shows that the monomer‐to‐excimer ratio increases upon stretching and scales almost linearly with the applied strain, which again supports the proposed force‐induced dissociation of the pyrene dimers.

We then evaluated the reversibility of the mechanochromic response of **PU‐CPy** through cycling tensile tests, which involved repeated stress‐release cycles. The polymer film was continuously stretched from 0% to 480% strain with a strain rate of 2.5% s^−1^, while recording the luminescence spectra. A correlation is observed between the 𝐼_470_/𝐼_580_ ratio and the applied strain for both stretch and release processes (Figure S18). However, some hysteresis was evident during the first two cycles, which is consistent with the known viscoelastic behavior of some polyurethane materials [30].

Long‐term reversibility was assessed by monitoring the monomer‐to‐excimer ratio over 30 consecutive cycles (strain rate 30% s^−1^) at two defined strain points, namely at 480% and 0% strain (Figure 4e). The emission response was generally well maintained throughout cycling. Admittedly, the first eight cycles exhibited a decrease of ca. 4.4% in the monomer‐to‐excimer ratio (defined as the percent ratio between the values of 𝐼_470_/𝐼_580_ in the eight and first cycle), but the loss appears significantly more contained starting from the tenth cycle (decrease of ca. 3.0% in cycles 10–30). This loss could originate from steric effects or noncovalent interactions between mechanophores and polymer chains that partially hinder the reformation of the dimers.

The polymer film was then quickly stretched (30% s^−1^ strain rate) and held at 480% strain for 300 s, while collecting the fluorescence response (Figure S19). The overlay of the stress‐strain curve and 𝐼_470_/𝐼_580_ ratio shows a first sharp increase in both stress and monomer‐to‐excimer ratio, consistent with the mechanoresponsive behavior in Figure 4d. However, when the polymer is held at a constant strain, the stress gradually decreases, while the 𝐼_470_/𝐼_580_ ratio continues increasing almost linearly. We hypothesized that photobleaching of the pyrene units in **CPy** might contribute to the observed spectral changes during the stress‐relaxation experiments [31, 32], as well as to the overall ca. 8% decrease in the monomer‐to‐excimer ratio in the cycling experiments discussed above. Thus, we carried out control experiments in which **PU‐CPy** films were exposed to continuous irradiation for 1 h, using the same 365 nm LED used for the experiments shown in Figure 4c. The measurements revealed that prolonged irradiation caused photobleaching of the excimer emission (Figure S20), which justifies the linear increase of the monomer‐to‐excimer ratio observed in the final stages of the stress‐relaxation experiments. While we acknowledge the photobleaching of **CPy** in **PU‐CPy**, we rule out the possibility of artefacts caused by photobleaching during the uniaxial experiments shown in Figure 4c. The two processes occur on significantly different timescales, with mechanical testing lasting approximately 2 min at a strain rate of 2.5% s^−1^, and photobleaching resulting from a continuous exposure to UV light for one hour.

To further substantiate this conclusion, tensile experiments on **PU‐CPy** films were conducted at varying strain rates (1% s^−1^ to 30% s^−1^), with corresponding total deformation times ranging from 480s to 16s. No significant differences in the 𝐼_470_/𝐼_580_ ratio were observed across the range of strain rates, further supporting the negligible role of photobleaching under experimental conditions (Figure S21). Taken together, these results strongly support that the reported fluorescence variation originates from the force‐induced dissociation of pyrene excimers within the calixarene‐based mechanophore, rather than from photodegradation processes.

The role of intramolecular interactions in the mechanoresponsive behavior of **PU‐CPy** was assessed by investigating the luminescence response of the control polymer **PU‐CmPy** under identical deformation conditions (0%–480% strain, 2.5% s^−1^ strain rate, *λ*_ex_ = 365 nm). In the absence of mechanical stress, the luminescence properties of **PU‐CmPy** resemble those of **CmPy** (Figure S22), showing no evidence of the excimer emission band that is observed in **PU‐CPy**. Only monomeric emission was observed throughout the entire stretching process, with no spectral changes either visually (Figure S23; Supplementary Movie S2) or spectroscopically (Figure 4f,g). Thus, we conclude that *inter*molecular interactions do not play a role in the dimerization process observed for **PU‐CPy**. This also supports the conclusion that the *cone* calixarene scaffold is crucial in promoting the *intra*molecular dimerization of the pyrene units.

Finally, we investigated the luminescence response of **0.025CPyMeinPU**, **0.05CPyMeinPU**, and **0.1CPyMeinPU** blends to mechanical stress. The three samples exhibited a more pronounced emission band at 580 nm (i.e., pyrene excimer) in the stress‐free state, compared to the covalently functionalized **PU‐CPy** (Figures 4h,i and S24), supporting the hypothesis that the covalent functionalization of **CPy** with polymer chains—as in **PU‐CPy**—influences the conformational equilibrium of the calixarene and partially hinders the dimerization of the pyrenes. Gratifyingly, no significant change in both the luminescence (Figure S25; Supporting Movie S3) and the emission profiles (Figure S24) was observed upon stretching under identical conditions (0%–480% strain, 2.5% s^−1^ strain rate, *λ*_ex_ = 365 nm), highlighting the key role of covalent incorporation of the mechanophore into the polymeric matrix for mechanotransduction.

## 3 Conclusion

A *cone* calix[4]arene, functionalized with two pyrene derivatives at the distal positions of the upper rim (**CPy**), was prepared and covalently incorporated into a linear polyurethane elastomer. Spectroscopic studies confirmed ground‐state intramolecular interactions between the pyrenes of **CPy**, leading to excimer emission upon excitation with UV light, both as an isolated molecule and when integrated into the linear polymer. Uniaxial tensile elongation of the polyurethane elastomer films causes the physical dissociation of the excimer, resulting in an increased contribution from monomer emission and, consequently, observable fluorescence changes both visually and spectroscopically. The mechanochemical activation is reversible, although a decrease in emission intensity ratio was observed. This behavior could be attributed to steric effects or non‐covalent interactions between the polymer chains and the mechanophores, as well as photobleaching of the pyrene fluorophores. We speculate that selecting fluorophores more resistant to continuous UV exposure will further validate this design concept.

Overall, we have demonstrated that the conformation of a flexible calix[4]arene can be modulated by mechanical force. *Cone* calix[4]arenes have proven to be promising scaffolds for designing mechanophores based on the ratiometric response between monomeric and excimeric emissive species. Their preorganized structure promotes interactions between judiciously chosen moieties positioned at the upper rim, while their inherent conformational flexibility enables the physical dissociation of the excimers triggered by mechanical force. The calixarene scaffold also maintains the proximity of these groups, allowing the interactions to be restored once the force is removed. While we have shown that this concept can promote reversible mechanochemical activation using supramolecular mechanophores, we are currently considering its broader applicability. Future research will focus on extending this approach to mechanically labile covalent bonds and on integrating well‐established calixarene functions, including host‐guest chemistry.

## Author Contributions

**José Augusto Berrocal**: conceptualization, supervision, resources, project administration, formal analysis, data curation, validation, methodology, visualization, writing – review & editing, writing – original draft, funding acquisition. **Lucia Visieri**: conceptualization, investigation, methodology, validation, visualization, software, formal analysis, writing – review & editing, writing – original draft. **Alessandro Casnati**: writing – review & editing. **Laura Baldini**: writing – review & editing, funding acquisition, conceptualization, supervision, formal analysis, data curation, resources, validation.

## Conflicts of Interest

The authors declare no conflicts of interest.

## Supporting information



The authors have cited additional references within the Supporting Information [33, 34, 35, 36, 37, 38, 39, 40, 41, 42, 43].
**Supporting File 1**: anie72367‐sup‐0001‐Movie1.mp4.

**Supporting File 2**: anie72367‐sup‐0002‐Movie2.mp4.

**Supporting File 3**: anie72367‐sup‐0003‐Movie3.mp4.

**Supporting File 4**: anie72367‐sup‐0004‐Data.zip.

**Supporting File 5**: anie72367‐sup‐0005‐SuppMat.docx.

## Data Availability

The data that supports the findings of this study are available in the Supporting Information of this article

## References

[1] A. Zinke and E. Ziegler , “Zur Kenntnis des Härtungsprozesses von Phenol‐Formaldehyd‐Harzen, VII. Mitteilung″ Berichte der Dtsch,” Chem Gesellschaft 74 (1941): 1729–1736, 10.1002/cber.19410741102.

[2] V. Böhmer , “Calixarenes, Macrocycles With (Almost) Unlimited Possibilities,” Angewandte Chemie (International ed in English) 34 (1995): 713–745, 10.1002/anie.199507131.

[3] K. S. J. Iqbal and P. J. Cragg , “Transmembrane Ion Transport by Calixarenes and Their Derivatives,” Dalton Transactions (2007): 26–32, 10.1039/B613867P. 17160170

[4] R. Cacciapaglia , A. Casnati , L. Mandolini , et al., “Catalysis of Diribonucleoside Monophosphate Cleavage by Water Soluble Copper(II) Complexes of Calix[4]Arene Based Nitrogen Ligands,” Journal of the American Chemical Society 128 (2006): 12322–12330, 10.1021/ja0632106. 16967984

[5] R. Cacciapaglia , A. Casnati , L. Mandolini , et al., “Di‐ and Trinuclear Zn^2+^ Complexes of Calix[4]arene Based Ligands as Catalysts of Acyl and Phosphoryl Transfer Reactions,” Journal of Organic Chemistry 70 (2005): 624–630, 10.1021/jo0487350. 15651810

[6] R. Cacciapaglia , L. Mandolini , and R. Salvio , “1.19 ‐Supramolecular Catalysis by Calixarenes,” Comprehensive Supramolecular Chemistry II (Elsevier, 2017): 459–478, 10.1016/B978-0-12-409547-2.05618-3.

[7] F. Sansone , L. Baldini , A. Casnati , and R. Ungaro , “Calixarenes: From Biomimetic Receptors to Multivalent Ligands for Biomolecular Recognition,” New Journal of Chemistry 34 (2010): 2715, 10.1039/c0nj00285b.

[8] A. Arduini , M. Fabbi , M. Mantovani , et al., “Calix[4]arenes Blocked in a Rigid Cone Conformation by Selective Functionalization at the Lower Rim,” The Journal of Organic Chemistry 60 (1995): 1454–1457, 10.1021/jo00110a055.

[9] M. Lazzarotto , F. Sansone , L. Baldini , A. Casnati , P. Cozzini , and R. Ungaro , “Synthesis and Properties of Upper Rim C‐Linked Peptidocalix[4]arenes,” European Journal of Organic Chemistry (2001): 595–602, 10.1002/1099-0690(200102)2001:3<595::AID-EJOC595>3.0.CO;2-.

[10] A. Arduini , S. Fanni , A. Pochini , A. R. Sicuri , and R. Ungaro , “Highly Distorted Cone Calix[4]arenes Through Intramolecular Mc Murry Coupling Reaction,” Tetrahedron 51 (1995): 7951–7958, 10.1016/0040-4020(95)00411-Z.

[11] O. Struck , J. P. M. Van Duynhoven , W. Verboom , S. Harkema , and D. N. Reinhoudt , “Cavity Effect of Calix[4]Arenes in Electrophilic Aromatic Substitution Reactions,” Chemical Communications 4 (1996): 1517, 10.1039/cc9960001517.

[12] P. F. Hudrlik , A. M. Hudrlik , L. Zhang , W. D. Arasho , and J. Cho , “Calix[4]arenes With Siloxanes Bridging Opposite Rings,” Journal of Organic Chemistry 72 (2007): 7858–7862, 10.1021/jo070661f. 17850096

[13] W. Hüggenberg , A. Seper , I. M. Oppel , and G. Dyker , “Multifold Photocyclization Reactions of Styrylcalix[4]arenes,” European Journal of Organic Chemistry (2010): 6786–6797, 10.1002/ejoc.201001108.

[14] J. A. Berrocal , M. B. Baker , L. Baldini , A. Casnati , and S. Di Stefano , “Inherently Chiral Cone‐Calix[4]Arenes via a Subsequent Upper rim Ring‐Closing/Opening Methodology,” Organic & Biomolecular Chemistry 16 (2018): 7255–7264, 10.1039/C8OB01813H. 30259046

[15] M. Galli , J. A. Berrocal , S. Di Stefano , et al., “Highly Efficient Intramolecular Cannizzaro Reaction Between 1,3‐distal Formyl Groups at the Upper Rim of a Cone‐calix[4]Arene,” Organic & Biomolecular Chemistry 10 (2012): 5109, 10.1039/c2ob25458a. 22618198

[16] E. Spatola , F. Rispoli , D. Del Giudice , et al., “Dissipative Control of the Fluorescence of a 1,3‐Dipyrenyl Calix[4]Arene in the Cone Conformation,” Organic & Biomolecular Chemistry 20 (2022): 132–138, 10.1039/D1OB02096J. 34816861

[17] B. Bardi , I. Tosi , F. Faroldi , et al., “A Calixarene‐Based Fluorescent Ratiometric Temperature Probe,” Chemical Communications 55 (2019): 8098–8101, 10.1039/C9CC04577E. 31232416

[18] M. Torelli , F. Terenziani , A. Pedrini , et al., “Mechanically‐Driven Vase–Kite Conformational Switch in Cavitand Cross‐Linked Polyurethanes,” ChemistryOpen 9 (2020): 261–268, 10.1002/open.201900345. 32128296 PMC7043258

[19] C. Löwe and C. Weder , “Oligo(p‐phenylene vinylene) Excimers as Molecular Probes: Deformation‐Induced Color Changes in Photoluminescent Polymer Blends,” Advanced Materials 14 (2002): 1625–1629, 10.1002/1521-4095(20021118)14:22<;1625::AID-ADMA1625>;3.0.CO;2-Q.

[20] H. Traeger , Y. Sagara , D. J. Kiebala , S. Schrettl , and C. Weder , “Folded Perylene Diimide Loops as Mechanoresponsive Motifs,” Angewandte Chemie International Edition 60 (2021): 16191–16199, 10.1002/anie.202105219. 33961723

[21] Y. Sagara , H. Traeger , J. Li , et al., “Mechanically Responsive Luminescent Polymers Based on Supramolecular Cyclophane Mechanophores,” Journal of the American Chemical Society 143 (2021): 5519–5525, 10.1021/jacs.1c01328. 33784073

[22] S. Shimizu , J. M. Clough , C. Weder , and Y. Sagara , “Hinge‐Like Mechanochromic Mechanophores Based on [2.2]Paracyclophane,” Angewandte Chemie International Edition 64 (2025): e202510114, 10.1002/anie.202510114. 40589206 PMC12416447

[23] Y. Sagara , M. Karman , E. Verde‐Sesto , et al., “Rotaxanes as Mechanochromic Fluorescent Force Transducers in Polymers,” Journal of the American Chemical Society 140 (2018): 1584–1587, 10.1021/jacs.7b12405. 29355316 PMC5806082

[24] E. McCann and M. Koshino , “The Electronic Properties of Bilayer Graphene,” Reports on Progress in Physics 76 (2013): 056503, 10.1088/0034-4885/76/5/056503. 23604050

[25] P. Reynders , W. Kuehnle , and K. A. Zachariasse , “Ground‐State Dimers in Excimer‐forming Bichromophoric Molecules. 1. bis(pyrenylcarboxy)Alkanes,” Journal of the American Chemical Society 112 (1990): 3929–3939, 10.1021/ja00166a032.

[26] R. G. Bryant , “The NMR Time Scale,” Journal of Chemical Education 60 (1983): 933, 10.1021/ed060p933.

[27] D. A. Van Dyke , B. A. Pryor , P. G. Smith , and M. R. Topp , “Nanosecond Time‐Resolved Fluorescence Spectroscopy in the Physical Chemistry Laboratory: Formation of the Pyrene Excimer in Solution,” Journal of Chemical Education 75 (1998): 615, 10.1021/ed075p615.

[28] M. Sauer , J. Hofkens , and J. Enderlein , Handbook of Fluorescence Spectroscopy and Imaging: From Ensemble to Single Molecules (VCH, 2011), 10.1002/9783527633500.

[29] T. Yamakado and S. Saito , “Ratiometric Flapping Force Probe That Works in Polymer Gels,” Journal of the American Chemical Society 144 (2022): 2804–2815, 10.1021/jacs.1c12955. 35108003

[30] C. Prisacariu , Polyurethane Elastomers (Springer, 2011), 10.1007/978-3-7091-0514-6.

[31] Y. P. Sun , B. Ma , G. E. Lawson , C. E. Bunker , and H. W. Rollins , “Effects of Photochemical Reactions Of Pyrene In Alcohol And Aqueous Solvent Systems On Spectroscopic Analyses,” Analytica Chimica Acta 319 (1996): 379–386, 10.1016/0003-2670(95)00507-2.

[32] J. N. Grossman , A. P. Stern , M. L. Kirich , and T. F. Kahan , “Anthracene and Pyrene Photolysis Kinetics in Aqueous, Organic, and Mixed Aqueous‐organic Phases,” Atmospheric Environment 128 (2016): 158–164, 10.1016/j.atmosenv.2015.12.049.

[33] G. Hennrich , M. T. Murillo , P. Prados , et al., “Alkynyl Expanded Donor–Acceptor Calixarenes: Geometry and Second‐Order Nonlinear Optical Properties,” Chemistry—A European Journal 13 (2007): 7753–7761, 10.1002/chem.200700615. 17610220

[34] W. H. Gardiner , M. Camilleri , L. A. Martinez‐Lozano , S. P. Bew , and G. R. Stephenson , “Upper‐Rim Monofunctionalisation in the Synthesis of Triazole‐ and Disulfide‐Linked Multicalix[4]‐ and ‐[6]arenes,” Chemistry – A European Journal 24 (2018): 19089–19097, 10.1002/chem.201804755. 30325070

[35] C. M. Payne , K. Cho , and D. S. Larsen , “5‐Bromo‐norborn‐2‐en‐7‐one Derivatives as a Carbon Monoxide Source for Palladium Catalyzed Carbonylation Reactions,” RSC Advances 9 (2019): 30736–30740, 10.1039/C9RA06594F. 35529407 PMC9072167

[36] B.‐J. Jeon , S. W. Cha , M.‐Y. Jeong , T. K. Lim , and J.‐I. Jin , “Synthesis and 2^nd^ Order Nonlinear Optical Properties Of Soluble Polyimides Bearing Nitroazobenzene Type Chromophore Pendants Attached In Side‐On,” Journal of Materials Chemistry 12 (2002): 546–552, 10.1039/b107553e.

[37] D. Stenkamp , S. G. Mueller , P. Lustenberger , et al., (2005), (Boehringer Ingelheim) US20050267115A1.

[38] A. Ouach , F. Pin , E. Bertrand , et al., “Design of α7 nicotinic acetylcholine receptor ligands using the (het)Aryl‐1,2,3‐triazole core: Synthesis, in vitro Evaluation and SAR Studies,” European Journal of Medicinal Chemistry 107 (2016): 153–164, 10.1016/j.ejmech.2015.11.001. 26580980

[39] J. Holub , V. Eigner , L. Vrzal , H. Dvoráková , and P. Lhoták , “Calix[4]Arenes With Intramolecularly Bridged Meta Positions Prepared via Pd‐Catalysed Double C–H Activation,” Chemical Communications 49 (2013): 2798–2800, 10.1039/C3CC40655E. 23440292

[40] F. Elaieb , A. Hedhli , D. Sémeril , D. Matt , and J. Harrowfield , “A Calixarene‐Decorated Phosphole Oxide,” European Journal of Organic Chemistry 2016 (2016): 3103–3108, 10.1002/ejoc.201600381.

[41] Rigaku , (2022), Empirical Absorption Correction Using Spherical Harmonics, Implemented In Scale3 Abspack Scaling Algorithm.

[42] G. M. Sheldrick , “SHELXT—Integrated Space‐Group And Crystal‐Structure Determination,” Acta Crystallographica Section A, Crystal Physics, Diffraction, Theoretical and General Crystallography 71 (2015): 3–8, 10.1107/S2053273314026370. PMC4283466 25537383

[43] O. V. Dolomanov , L. J. Bourhis , R. J. Gildea , J. A. K. Howard , and H. Puschmann , “OLEX2: A Complete Structure Solution, Refinement And Analysis Program,” Journal of Applied Crystallography 42 (2009): 339–341, 10.1107/S0021889808042726.

