# Supplementary material for: Mechanically Activated Luminescence in Polyurethanes Incorporating Calixarene Mechanophores
Source: Angew Chem Int Ed Engl. 2026 Apr 27;65(24):e9927716. doi: 10.1002/anie.9927716 (PMC13245617; doi:10.1002/anie.9927716)
Supplement: Supplementary file 5 — Supporting File 5: anie72367‐sup‐0005‐SuppMat.docx. [file ANIE-65-e9927716-s005.docx]

Supporting Information for

Mechanically Activated Luminescence in Polyurethanes Incorporating Calixarene Mechanophores

Lucia Visieri,^[a]^ Alessandro Casnati,^[b]^ Laura Baldini,*^[b]^ and José Augusto Berrocal*^[a], [c]^

[a] Institute of Chemical Research of Catalonia (ICIQ), The Barcelona Institute of Science and Technology (BIST), Av. Països Catalans 16, 43007 Tarragona (Spain)

[b] Dipartimento di Scienze Chimiche, della Vita e della Sostenibilità Ambientale, Università degli Studi di Parma, Parco Area delle Scienze 17/a, 43124 Parma (Italy)

[c] Catalan Institution for Research and Advanced Studies (ICREA), Pg. Lluís Companys 23, 08010 Barcelona (Spain)

* to whom correspondence should be addressed: [laura.baldini@unipr.it](mailto:laura.baldini@unipr.it); jberrocal@iciq.es

**Contents**

[General Materials and Methods 3](#_Toc221613395)

[Materials 3](#_Toc221613396)

[Instrumentations and Methods 3](#_Toc221613397)

[Synthetic Procedures and Analytical Data 5](#_Toc221613398)

[Polymer synthesis 11](#_Toc221613399)

[Thermal properties 13](#_Toc221613400)

[Mechanical properties 16](#_Toc221613401)

[Solid-state crystal structure of CPy 17](#_Toc221613402)

[^1^H NMR spectra of CPy, CmPy, and CPyMe 18](#_Toc221613403)

[DOSY spectrum of CPy 19](#_Toc221613404)

[Emission spectrum of CPyMe 20](#_Toc221613405)

[Concentration dependency of luminescence spectra of CPy and CmPy 20](#_Toc221613406)

[Emission decays of CPy and CPyMe 21](#_Toc221613407)

[Photophysical properties of PU-CPy in solution 22](#_Toc221613408)

[Stress-strain curves for two stress and release cycles 23](#_Toc221613409)

[Fluorescence intensity ratio during stress relaxation 23](#_Toc221613410)

[Fluorescence intensity ratio during one-hour continuous irradiation 24](#_Toc221613411)

[Stress and fluorescence response at different strain rates 25](#_Toc221613412)

[Luminescence properties of PU-CmPy 27](#_Toc221613413)

[Luminescence of PU-CmPy upon elongation 27](#_Toc221613414)

[Luminescence properties and correlation between stress-strain curves and the fluorescence intensity ratio of CPyMe blends in PU upon stretching 28](#_Toc221613415)

[Luminescence of 0.025CPyMeinPU upon elongation 29](#_Toc221613416)

[NMR spectra 29](#_Toc221613417)

Single crystal X-Ray Diffraction…………………………………………………………………………………………………………………….39

# General Materials and Methods

# Materials

1,4-butanediol was distilled under vacuum (140 °C, 10^-5^ mbar) immediately prior to use. Poly(tetrahydrofuran) was dried at 80 °C under vacuum (10^-5^ mbar) for 18 hours prior to use. Other commercially available reagents and solvents were used without any further purification or treatment, except when explicitly stated. All moisture- and air-sensitive reactions were conducted under a nitrogen or argon atmosphere, using previously oven-dried glassware. Dry solvents were obtained by the Innovative Technology Solvent Purification System or prepared according to standard procedures, and stored under 3 or 4 Å molecular sieves. Monitoring of synthetic processes was performed using direct-phase thin-layer chromatography (TLC) on 60 F254 silica gel plates on aluminium from Merck. Revelation of reagents and products was achieved with UV light (254 and 365 nm) or using staining agents. For the detection of easily oxidizable compounds, the TLCs were sprayed with a 0.05 wt% solution of KMnO_4_ in water. For compounds with phenolic groups, a 1 wt% solution of FeCl_3_ in a 1:1 water/methanol mixture was used. Merck silica gel 60 (230-400 mesh) was used to perform flash chromatography purifications under air pressure.

# Instrumentations and Methods

**Nuclear magnetic resonance (NMR) spectroscopy.** NMR spectra were recorded with a Bruker AVANCE 400 spectrometer at 298 K. ^1^H NMR spectra were recorded at 400 MHz, while ^13^C NMR spectra were recorded at 100 MHz and with proton decoupling. Low-temperature ^1^H NMR spectra were recorded at 500 MHz with a Bruker AVANCE 500 spectrometer equipped with a cryoprobe. Spectra were calibrated to the residual solvent peak of CDCl_3_. Chemical shift values ($\delta$) are reported in ppm using the resonance frequency of the partially deuterated solvent as an internal standard. The coupling constants (*J*) are reported in Hertz. The multiplicity is expressed as follows: s = singlet, d = doublet, t = triplet, ses = sestuplet, m = multiplet, bs = broad signal.

**Mass spectroscopy (MS).** Mass spectra were recorded using electrospray ionization (ESI) in positive mode with a single quadrupole instrument SQ Detector, Waters (capillary voltage 3.7 kV, cone voltage 30–160 eV, extractor voltage 3eV, source block temperature 80°C, desolvation temperature 150°C, cone and desolvation gas (N2) flow rates 1.6 and 8 L/min, respectively). MeOH was used as a solvent.

**UV-vis absorption spectroscopy.** UV-Vis absorption spectra were recorded on a Thermo Scientific Evolution 260 Bio spectrophotometer or a PerkinElmer Lambda 650 spectrophotometer.

**Fluorescence spectroscopy.** Steady-state fluorescence and fluorescence decay measurements were carried out on an Edinburgh FLS1000 spectrofluorometer using a 450 W Xenon Lamp as a source and a PMT-900 detector. Fluorescence spectra are corrected for the excitation intensity and the detector sensitivity. For fluorescence quantum yield measurements, fluorescein in NaOH 0.1 M was used as a reference (quantum yield 0.91). Fluorescence lifetime measurements were performed using 405 nm ps pulsed diode lasers.

**Compression molding.** Polymer films were prepared by compression molding in a Carver Bench Top Laboratory Manual Press with Electrically Heated Platens (Model 4386) at a temperature of 120 ºC between two poly(tetrafluoroethylene) (PTFE) sheets with PTFE spacers with a thickness of ca. 0.25 mm. The pressure was gradually increased over 5 cycles (0.80 tons increase per cycle), reaching a maximum of 4 tons, with each cycle lasting approximately 20 seconds. The films were subjected to maximum pressure and temperature for 3 minutes, and then slowly cooled to room temperature over a two-hour period while being held between the metal plates.

**Tensile testing.** Tensile testing of the polymer films was done using a Linkam Modular Force Stage (MFS) equipped with a 200 N force cell. Uniaxial tensile tests were carried out with rectangular samples with dimensions of 40 x 5 x 0.2 mm (length x width x thickness) at room temperature and with a strain rate of 2.5 % (unless otherwise specified). Young’s moduli were calculated from the slope in the linear region between 0 to 5 % strain.

**In situ fluorescence measurements.** The emission spectra of the polymer films upon tensile elongation were collected with an Ocean Insight spectrophotometer coupled with an Ocean Optics LDC-1C LED Controller.

**Differential scanning calorimetry (DSC).** DSC measurements were conducted on a Mettler Toledo DSC822e STAR system under nitrogen, in a temperature range from -80 to 200 °C, using a heating and cooling rate of 10 °C min^-1^. An isothermal hold of 5 min was applied at the end of each heating and cooling cycle. The shown data come from the first cooling run and the second heating run.

**Thermogravimetric analysis (TGA).** TGA analysis was conducted on a Mettler Toledo TGA/SDTA851 STAR system under nitrogen, in a temperature range from 25 to 600 °C, with a heating rate of 10 °C min^-1^.

**Size exclusion chromatography (SEC).** SEC experiments were performed on an Agilent 1200 series HPLC system equipped with a PSSSDV Analytical linear M GPC column (8 x 300 mm, particle size = 5 μm). Samples were run using THF as the eluent at 30 °C at a flow rate of 1.0 mL min^−1^. Samples were analysed at a concentration of 1 mg mL^−1^ after filtration through a 0.45 μm pore-size membrane. Molecular weights and dispersion were derived from the RI signal using a calibration curve based on polystyrene standards (PS from Polymer Standards Service).

**Photographs.** Photographs and videos of manually deformed polymer films under UV illumination (365 nm) were recorded using a Sony Alpha 6000 digital camera equipped with an E PZ 16-50 mm lens f/3.5-5.6 OSS in manual focus mode.

# Synthetic Procedures and Analytical Data

**Scheme S1.** Synthesis of calixarene **3**.

**5,17-diiodo-25,27-dipropoxycalix[4]arene (1a).** In a 100 mL two-necked round-bottom flask, under a N_2_ atmosphere, 25,27-dipropoxycalix[4]arene (1.0 g, 2.0 mmol) was dissolved in chloroform (50 mL). AgOTFA (1.7 g, 7.8 mmol) was added, and the mixture was stirred at 65 °C for 15 minutes. I_2_ (2.0 g, 7.8 mmol) was then added, and the suspension was heated at 65 °C under magnetic stirring for 1 hour and 45 minutes. The crude was filtered over celite to remove solid AgI, and the organic phase was washed with Na_2_S_2_O_3_ (3 x 50 mL) and water. The solvent was dried over Na_2_SO_4_ and evaporated under reduced pressure. Due to solubility issues, the crude material was not purified.

**5,17-diiodo-25,26,27,28-tetrapropoxycalix[4]arene (2).** In a 100 mL two-necked round-bottom flask, under a N_2_ atmosphere, compound **1** (1.2 g, 2.0 mmol) was dissolved in dry DMF (30 mL). NaH 60 wt% (0.47 g, 12 mmol) and *n*-iodopropane (0.77 mL, 7.8 mmol) were added. The mixture was stirred at room temperature for 72 hours and then quenched by adding 1 M HCl (30 mL) to the reaction mixture. The solid precipitate was filtered and washed with methanol. Column chromatography purification (silica gel, hexane/DCM 95:5–85:15 v/v) afforded the product as a white solid (0.17 g, 0.20 mmol) in 11% yield. ^1^H NMR (CDCl_3_, 400 MHz) δ (ppm): 7.14 (s, 4H, ArH), 6.65 – 6.43 (m, 6H, ArH), 4.39 (d, *J* = 13.4 Hz, 4H, ArCH*H_ax_*Ar), 3.89 (t, *J* = 7.3 Hz, 4H, O*CH_2_*CH_2_CH_3_), 3.79 (t, *J* = 7.3 Hz, 4H, O*CH_2_*CH_2_CH_3_), 3.11 (d, *J* = 13.4 Hz, 4H, ArCH*H_eq_*Ar), 2.00 – 1.77 (m, 8H, OCH_2_*CH_2_*CH_3_), 1.09 – 0.93 (m, 12H, OCH_2_CH_2_*CH_3_*).

The physical and chemical properties of **2** are consistent with those reported in the literature.^[33]^

**5,17-bis(trimethylsilylethynyl)-25,26,27,28-tetrapropoxycalix[4]arene (2a).** A 25 mL Schlenk tube was charged with compound **4** (0.17 g, 0.20 mmol), Pd(PPh_3_)_2_Cl_2_ (8.0 mg, 0.012 mmol), and CuI (3.0 mg, 0.017 mmol). Three vacuum/nitrogen cycles were applied. Under a N_2_ atmosphere, dry THF (2 mL), TMSA (0.10 mL, 0.71 mmol), and dry triethylamine (1 mL) were added. The mixture was stirred at 65 °C for 18 hours and then diluted with DCM (30 mL). The organic phase was washed with 0.1 M HCl (2 x 30 mL) and water (30 mL), and dried over Na_2_SO_4_. The solvent was removed under reduced pressure, and the crude was purified by column chromatography (silica gel, hexane/DCM 98:2–90:10 v/v). The product was obtained as a white solid (0.14 g, 0.18 mmol) in 89% yield. ^1^H NMR (CDCl_3_, 400 MHz) δ (ppm): 7.30 (s, 4H, ArH), 6.23 (t, *J* = 7.4 Hz, 2H, ArH), 6.15 (d, *J* = 7.5 Hz, 4H, ArH), 4.40 (d, *J* = 13.3 Hz, 4H, ArCH*H_ax_*Ar), 4.04 (t, *J* = 8.2 Hz, 4H, O*CH_2_*CH_2_CH_3_), 3.66 (t, *J* = 6.8 Hz, 4H, O*CH_2_*CH_2_CH_3_), 3.13 (d, *J* = 13.3 Hz, 4H, ArCH*H_eq_*Ar), 1.98 – 1.83 (m, 8H, OCH_2_*CH_2_*CH_3_), 1.11 (t, *J* = 7.4 Hz, 6H, OCH_2_CH_2_*CH_3_*), 0.89 (t, *J* = 7.5 Hz, 6H, OCH_2_CH_2_*CH_3_*), 0.30 (s, 18H, SiCH_3_). ^13^C NMR (100 MHz, CDCl_3_) δ (ppm): 158.7, 154.9, 137.1, 132.6, 132.4, 127.6, 122.2, 116.0, 105.9, 92.3, 76.5, 30.7, 23.5, 22.9, 10.8, 9.8. ESI-MS: m/z calcd for C_50_H_64_O_4_Si_2_ [(**3**+H)^+^] 785.4, found 785.6 (40%); calcd for C_50_H_64_O_4_Si_2_Na [(**3**+Na)^+^] 807.4, found 807.6 (100%); calcd for C_50_H_64_O_4_Si_2_K [(**3**+K)^+^] 823.5, found 824.5 (65%).

**5,17-diethynyl-25,26,27,28-tetrapropoxycalix[4]arene (3).** In a 50 mL round-bottom flask, compound **3** (0.20 g, 0.25 mmol) was suspended in 20 mL of methanol. K_2_CO_3_ (0.18 g, 1.3 mmol) was added. The reaction mixture was stirred at room temperature for 24 hours, and the solvent was removed under reduced pressure. The mixture was dissolved in DCM (20 mL) and then washed with 1 M HCl (20 mL), water (20 mL), and brine (20 mL). The organic phase was dried over Na_2_SO_4_, and the solvent was removed under reduced pressure. The product was obtained as a pale yellow solid (163 mg, 0.21 mmol) in 83% yield. ^1^H NMR (CDCl_3_, 400 MHz) δ (ppm): 7.10 (s, 4H, ArH), 6.47 – 6.34 (m, 6H, ArH), 4.43 (d, *J* = 13.4 Hz, 4H, ArCH*H_ax_*Ar), 3.97 (t, *J* = 7.9 Hz, 4H, O*CH_2_*CH_2_CH_3_), 3.75 (t, *J* = 6.3 Hz, 4H, O*CH_2_*CH_2_CH_3_), 3.15 (d, *J* = 13.4 Hz, 4H, ArCH*H_eq_*Ar), 2.98 (s, 2H, C≡CH), 1.85 – 2.00 (m, 8H, OCH_2_*CH_2_*CH_3_), 1.06 (t, *J* = 7.4 Hz, 6H, OCH_2_CH_2_*CH_3_*), 0.95 (t, *J* = 7.5 Hz, 6H, OCH_2_CH_2_*CH_3_*).

The physical and chemical properties of **3** are consistent with those reported in the literature.^[34]^

**Scheme S2.** Synthesis of intermediate **4**.

**Ethyl 2-(4-iodophenoxy)acetate (5).** In a 50 mL two-necked round-bottom flask, under a N_2_ atmosphere, commercially available 4-iodophenol (2.0 g, 9.1 mmol) was dissolved in 15 mL of dry acetone. K_2_CO_3_ (1.5 g, 14 mmol) and ethyl bromoacetate (1.5 mL, 14 mmol) were added under positive N_2_ flow. The mixture was heated to reflux for 24 hours. The mixture was diluted with DCM (50 mL) and washed with 1 M HCl (2 x 50 mL) and brine (50 mL). The solvent was removed under reduced pressure, and the crude was purified by column chromatography (silica gel, hexane/AcOEt 9:1 v/v). The product was obtained as a white solid (2.7 g, 8.7 mmol) in 95% yield. ^1^H NMR (CDCl_3_, 400 MHz) δ (ppm): 7.59 (d, *J* = 8.9 Hz, 2H, ArH), 6.71 (d, *J* = 8.9 Hz, 2H, ArH), 4.61 (s, 2H, CH_2_CO), 4.29 (q, *J* = 7.1 Hz, 2H, *CH_2_*CH_3_), 1.32 (t, *J* = 7.1 Hz, 3H, CH_2_*CH_3_*).

The physical and chemical properties of **4** are consistent with those reported in the literature.^[35]^

**2-(4-iodophenoxy)ethan-1-ol (6).** In a 100 mL two-necked round-bottom flask, a solution of compound **5** (2.7 g, 8.7 mmol) in dry THF (30 mL) was cooled to -10 °C. A suspension of LiAlH_4_ (0.7 g, 17 mmol) in dry THF (10 mL) was added dropwise. The mixture was stirred at -10 °C for 30 minutes, and then at room temperature for 15 minutes. The reaction was quenched by adding 1 M HCl (30 mL) dropwise. The mixture was extracted with DCM (3 x 40 mL). The combined organic phases were washed with brine, dried over Na_2_SO_4_, and evaporated under reduced pressure to afford the product as a white solid (1.6 g, 6.1 mmol) in 70% yield. ^1^H NMR (CDCl_3_, 400 MHz) δ (ppm): 7.59 (d, *J* = 8.9 Hz, 2H, ArH), 6.73 (d, *J* = 8.9 Hz, 2H, ArH), 4.07 (t, *J* = 4.9 Hz, 2H, *CH_2_*CH_2_OH), 3.98 (t, *J* = 4.9 Hz, 2H, CH_2_*CH_2_*OH), 2.00 (bs, 1H, OH).

The physical and chemical properties of **6** are consistent with those reported in the literature.^[36]^

**Synthesis of 2-(4-((trimethylsilyl)ethynyl)phenoxy)ethan-1-ol (7).** In a 100 mL Schlenk tube, under a N_2_ atmosphere, compound **6** (1.6 g, 6.1 mmol), Pd(PPh_3_)_2_Cl_2_ (43 mg, 0.061 mmol), and CuI (34 mg, 0.18 mmol) were suspended in dry THF (6 mL). Triethylamine (2 mL) and trimethylsilylacetylene (1.1 mL, 7.9 mmol) were then added. The mixture was stirred at 40 °C for 18 hours and quenched by opening the reaction vessel to air. The reaction mixture was diluted with ethyl acetate (40 mL), and the organic phase was washed with 1 M HCl (3 x 30 mL) and brine (30 mL). The organic phase was dried over Na_2_SO_4_ and evaporated under reduced pressure. After purification by column chromatography (silica gel, hexane/ethyl acetate 9:1–7:3 v/v), the product was obtained in 97% yield as a pale-yellow solid (1.4 g, 5.9 mmol). ^1^H NMR (CDCl_3_, 400 MHz) δ (ppm): 7.43 (d, *J* = 8.8 Hz, 2H, ArH), 6.85 (d, *J* = 8.8 Hz, 2H, ArH), 4.09 (t, *J* = 4.8 Hz 2H, *CH_2_*CH_2_OH), 3.98 (bs, 2H, CH_2_*CH_2_*OH), 2.14 (bs, 1H, OH), 0.26 (s, 9H, CH_3_).

The physical and chemical properties of **7** are consistent with those reported in the literature.^[37]^

**Synthesis of 2-(4-ethynylphenoxy)ethan-1-ol (8).** In a 50 mL round-bottom flask, compound **7** (0.36 g, 1.6 mmol) was dissolved in methanol (20 mL). K_2_CO_3_ (0.67 g, 4.8 mmol) was added. After 18 hours of stirring at room temperature, the reaction mixture was diluted with DCM (50 mL) and washed with 1 M HCl (2 x 40 mL) and brine (40 mL). The organic phase was dried over Na_2_SO_4_, and the solvent was removed under reduced pressure, providing a pale brown solid (0.24 g, 1.5 mmol) with 94% yield. ^1^H NMR (CDCl_3_, 400 MHz) δ (ppm): 7.46 (d, J = 8.8 Hz, 2H, ArH), 6.88 (d, *J* = 8.8 Hz, 2H, ArH), 4.11 (d, *J* = 4.9 Hz, 2H, *CH_2_*CH_2_OH), 3.99 (m, 2H, CH_2_*CH_2_*OH), 3.03 (s, 1H, CH), 2.06 (bs, 1H, OH).

The physical and chemical properties of **8** are consistent with those reported in the literature.^[38]^

**Compound 4.** Freshly prepared compound **8** (0.24 g, 1.5 mmol), 1,6-dibromopyrene (1.6 g, 4.5 mmol), Pd(PPh_3_)_2_Cl_2_ (53 mg, 0.075 mmol), and CuI (20 mg, 0.11 mmol) were suspended in a 100 mL Schlenk tube under a N_2_ atmosphere, using a 5:1 mixture of dry THF and dry triethylamine (12 mL). After 18 hours of stirring at 65 °C, the solvent was evaporated. The crude product was purified by column chromatography (silica gel, DCM). Compound **8** was obtained as a yellow solid (0.42 g, 0.95 mmol) in 63% yield. ^1^H NMR (CDCl_3_, 400 MHz) δ (ppm): 8.71 (d, *J* = 9.1 Hz, 1H, H_py_), 8.48 (d, *J* = 9.2 Hz, 1H, H_py_), 8.28 (d, *J* = 8.2 Hz, 1H, H_py_), 8.24 (d, *J* = 8.0 Hz, 1H, H_py_), 8.22 – 8.13 (m, 3H, H_py_), 8.08 (d, *J* = 8.2 Hz, 1H, H_py_), 7.69 (d, *J* = 8.9 Hz, 2H, ArH), 7.01 (d, *J* = 8.9 Hz, 2H, ArH), 4.19 (d, *J* = 4.8 Hz, 2H, *CH_2_*CH_2_OH), 4.07 – 4.02 (m, 2H, *CH_2_*OH), 2.04 (t, *J* = 5.9 Hz, 1H). ^13^C NMR (CDCl_3_, 100 MHz) δ (ppm): 158.92, 133.28, 131.75, 130.84, 130.61, 130.45, 130.09, 129.87, 128.85, 127.92, 126.58, 126.02, 125.97, 125.65, 125.11, 124.00, 120.50, 119.07, 115.99, 114.77, 95.60, 87.33, 69.31, 61.45. ESI-MS m/z calcd for C_26_H_17_BrO_2_: [M+H]^+^ 441.0, 443.0, found 441.4, 443.5.

**Scheme S3.** Synthesis of calixarene **12**.

**5-iodo-25,26,27,28-tetrapropoxycalix[4]arene** **(10)**. In a 100 mL two-necked round-bottom flask under a N_2_ atmosphere, 25,26,27,28-tetrapropoxycalix[4]arene **9** (0.30 g, 0.51 mmol) was dissolved in dry chloroform (50 mL). AgOTFA (0.037 g, 0.17 mmol) was added, and the mixture was stirred at 65 °C for 15 minutes. I_2_ (0.043 g, 0.17 mmol) was added, and the mixture was stirred for another 2 hours at 65 °C. The reaction mixture was filtered over celite to remove AgI. The organic phase was washed with 0.5 M Na_2_S_2_O_3_ (2 x 50 mL) and water (50 mL), and dried over Na_2_SO_4_. The solvent was evaporated under reduced pressure, and the crude material was purified by column chromatography (silica gel, hexane/DCM 95:5–90:10 v/v). The product was obtained as a white solid (0.11 g, 0.16 mmol) in 91% yield. ^1^H NMR (CDCl_3_, 400 MHz) δ (ppm): 6.83 – 6.69 (m, 9H, ArH), 6.50 (d, *J* = 7.5 Hz, 2H, ArH), 4.50 (d, *J* = 13.4 Hz, 2H, ArCH*H_ax_*Ar), 4.42 (d, *J* = 13.4 Hz, 2H, ArCH*H_ax_*Ar), 3.99 – 3.77 (m, 8H, O*CH_2_*CH_2_CH_3_), 3.21 (d, *J* = 13.4 Hz, 2H, ArCH*H_eq_*Ar), 3.12 (d, *J* = 13.6 Hz, 2H, ArCH*H_eq_*Ar), 2.01 – 1.86 (m, 8H, OCH_2_*CH_2_*CH_3_), 1.11 – 1.02 (m, 6H, OCH_2_CH_2_*CH_3_*), 0.99 (t, *J* = 7.5 Hz, 6H, OCH_2_CH_2_*CH_3_*).

The physical and chemical properties of **10** are consistent with those reported in the literature.^[39]^

**5-trimethylsilylethynyl-25,26,27,28-tetrapropoxycalix[4]arene (11)**. A 25 mL Schlenk tube was charged with compound **10** (0.11 mg, 0.16 mmol), Pd(PPh_3_)_2_Cl_2_ (16 mg, 0.023 mmol), and CuI (4.5 mg, 0.023 mmol). Three vacuum/N_2_ cycles were performed. Under positive N_2_ flux, dry THF (2 mL), dry triethylamine (2 mL), and TMSA (0.086 mL, 0.62 mmol) were added. The mixture was stirred for 18 hours at 65 °C. The reaction mixture was diluted with DCM (40 mL) and washed with 0.1 M HCl (2 x 40 mL) and water (40 mL). The solvent was dried over Na_2_SO_4_ and evaporated under reduced pressure. The crude material was purified by column chromatography (silica gel, hexane/DCM 95:5–90:10 v/v), affording **11** as a colourless oil (0.085 g, 0.12 mmol) in 79% yield. ^1^H NMR (CDCl_3_, 400 MHz) δ (ppm): 6.94 (s, 2H, ArH), 6.83 – 6.72 (m, 3H, ArH), 6.46 (s, 6H, ArH), 4.46 (d, *J* = 13.4 Hz, 2H, ArCH*H_ax_*Ar), 4.42 (d, *J* = 13.4 Hz, 2H, ArCH*H_ax_*Ar), 3.97 – 3.88 (m, 4H, O*CH_2_*CH_2_CH_3_), 3.85 – 3.72 (m, 4H O*CH_2_*CH_2_CH_3_), 3.17 (d, *J* = 13.4 Hz, 2H, ArCH*H_eq_*Ar), 3.13 (d, *J* = 13.4 Hz, 2H, ArCH*H_eq_*Ar), 2.02 – 1.84 (m, 8H, OCH_2_*CH_2_*CH_3_), 1.04 (t, *J* = 7.5 Hz, 6H, OCH_2_CH_2_*CH_3_*), 1.00 – 0.93 (m, 6H, OCH_2_CH_2_*CH_3_*), 0.25 (s, 9H, SiCH_3_).

The physical and chemical properties of **11** are consistent with those reported in the literature.^[40]^

**5-ethynyl-25,26,27,28-tetrapropoxycalix[4]arene** **(12)**. In a 50 mL round-bottom flask, compound **11** (0.085 g, 0.12 mmol) was dissolved in DCM (15 mL). Methanol (5 mL) and K_2_CO_3_ (0.051 g, 0.37 mmol) were added. The mixture was stirred at room temperature for 18 hours. The solvent was removed under reduced pressure. The crude product was dissolved in DCM (30 mL) and washed with 0.1 M HCl (2 x 50 mL) and water (50 mL). The solvent was dried over Na_2_SO_4_ and removed under reduced pressure. The product was obtained as a colourless solid (0.079 g, 0.13 mmol) in a quantitative yield. ^1^H NMR (CDCl_3_, 400 MHz) δ (ppm): 6.75 (s, 2H, ArH), 6.71 – 6.55 (m, 9H, ArH), 4.47 (d, *J* = 13.4 Hz, 2H, ArCH*H_ax_*Ar), 4.44 (d, *J* = 13.4 Hz, 2H, ArCH*H_ax_*Ar), 3.92 – 3.81 (m, 8H, O*CH_2_*CH_2_CH_3_), 3.18 (d, *J* = 13.4 Hz, 2H, ArCH*H_eq_*Ar), 3.14 (d, *J* = 13.4 Hz, 2H, ArCH*H_eq_*Ar), 2.89 (s, 1H, CH), 2.01 – 1.86 (m, 8H, OCH_2_*CH_2_*CH_3_), 1.11 – 0.92 (m, 12H, OCH_2_CH_2_*CH_3_*).

The physical and chemical properties of **12** are consistent with those reported in the literature.^[40]^

**Compound CPy.** A 50 mL Schlenk flask was charged with compound **4** (66 mg, 0.10 mmol), compound **9** (92 mg, 0.21 mmol), Pd(PPh_3_)_2_Cl_2_ (4.0 mg, 0.0052 mmol), and CuI (1.5 mg, 0.0073 mmol). After three vacuum/N_2_ cycles, dry triethylamine (1 mL) and dry DMF (5 mL) were added under positive N_2_ flux. The reaction mixture was stirred at 40 °C for 24 hours. 0.1 M HCl (30 mL) was added to quench the reaction. The crude material was diluted with DCM (30 mL). The organic phase was washed with brine (3 x 30 mL), dried over Na_2_SO_4_, and evaporated. **CPy** was obtained after purification by column chromatography (silica gel, DCM/acetone 100:0–90:10 v/v) as a yellow solid (64 mg, 0.047 mmol) in 45% yield. ^1^H NMR (CDCl_3_, 400 MHz) δ (ppm): 8.18 (d, *J* = 9.0 Hz, 2H, H_py_), 8.09 (d, *J* = 9.0 Hz, 2H, H_py_), 7.91 (d, *J* = 7.9 Hz, 2H, H_py_), 7.70 (d, *J* = 8.0 Hz, 2H, H_py_), 7.63 (d, *J* = 9.1 Hz, 2H, H_py_), 7.61 – 7.55 (m, 6H, H_ph_, H_py_), 7.48 (d, *J* = 9.0 Hz, 2H, H_py_), 7.36 (d, *J* = 8.0 Hz, 2H, H_py_), 7.15 (d, *J* = 7.5 Hz, 4H, ArH), 7.03 (t, *J* = 7.5 Hz, 2H, ArH), 6.84 (d, *J* = 8.7 Hz, 4H, H_ph_), 6.79 (s, 4H, ArH), 4.56 (d, *J* = 13.4 Hz, 4H, ArCH*H_ax_*Ar), 4.12 (t, *J* = 4.8 Hz, 4H, *CH_2_*CH_2_OH), 4.08 (t, *J* = 7.9 Hz, 4H, *OCH_2_*CH_2_CH_3_), 4.04 (t, *J* = 4.8 Hz, 4H, CH_2_*CH_2_*OH), 3.83 (t, *J* = 6.9 Hz, 4H, O*CH_2_*CH_2_CH_3_), 3.29 (d, *J* = 13.4 Hz, 4H, ArCH*H_eq_*Ar), 2.05 (q, *J* = 7.7 Hz, 4H, OCH_2_*CH_2_*CH_3_), 1.97 (q, *J* = 7.2 Hz, 6H, OCH_2_*CH_2_*CH_3_), 1.13 (t, *J* = 7.4 Hz, 6H, OCH_2_CH_2_*CH_3_*), 1.01 (t, *J* = 7.4 Hz, 6H, OCH_2_CH_2_*CH_3_*). ^13^C NMR (CDCl_3_, 100 MHz) δ (ppm): 158.48, 157.44, 156.40, 136.02, 134.42, 133.23, 131.28, 131.24, 130.83, 130.37, 129.61, 129.02, 128.96, 128.75, 127.16, 126.78, 125.80, 125.14, 124.11, 124.01, 123.23, 122.97, 122.24, 118.70, 117.84, 117.27, 116.51, 114.56, 96.06, 94.87, 87.89, 86.86, 77.08, 76.79, 69.40, 61.43, 30.98, 23.47, 23.12, 10.68, 10.06. ESI-MS m/z calcd for C_96_H_80_O_8_: [M+H]^+^ 1361.6, found 1362.0. ε_max_: 95506 M^-1^cm^-1^. Φ(380 nm): 42%.

**Scheme S4.** Synthesis of calixarene **CPyMe**.

**Compound CPyMe.** In a 25 mL two-necked round-bottom flask, compound **CPy** (24 mg, 0.018 mmol) was dispersed in dry DMF (6 mL) under a N_2_ atmosphere. NaH (4.2 mg, 0.11 mmol) and iodomethane (4.4 $\mu$L, 0.071 mmol) were added. The mixture was stirred for 18 hours at room temperature. 0.1 M HCl (20 mL) was added to quench the reaction. The mixture was extracted with DCM (2 x 20 mL). The combined organic layers were washed with brine (2 x 40 mL), dried over Na_2_SO_4_, and the solvent was evaporated under reduced pressure. **CPyMe** was obtained after purification by column chromatography (silica gel, DCM/hexane 10:0–9:1 v/v) as a yellow solid (20 mg, 0.014 mmol) in 80% yield. ^1^H NMR (CDCl_3_, 400 MHz) δ (ppm): 8.28 (d, *J* = 9.0 Hz, 2H, H_py_), 8.20 (d, *J* = 9.0 Hz, 2H, H_py_), 8.02 (d, *J* = 7.9 Hz, 2H, H_py_), 7.82 (d, *J* = 8.0 Hz, 2H, H_py_), 7.72 (d, *J* = 8.7 Hz, 2H, H_py_), 7.70 (d, *J* = 7.6 Hz, 2H, H_py_), 7.65 (d, *J* = 8.8 Hz, 4H, H_ph_), 7.62 (d, *J* = 9.1 Hz, 2H, H_py_), 7.51 (d, *J* = 8.0 Hz, 2H, H_py_), 7.04 – 6.87 (m, 14H, ArH, H_ph_), 4.56 (d, *J* = 13.4 Hz, 4H, ArCH*H_ax_*Ar), 4.18 (t, *J* = 4.8 Hz, 4H, OCH_2_*CH_2_*O), 4.02 (t, *J* = 7.7 Hz, 4H, *OCH_2_*CH_2_CH_3_), 3.90 (t, *J* = 7.2 Hz, 4H, *OCH_2_*CH_2_CH_3_), 3.82 (t, *J* = 4.8 Hz, 4H, O*CH_2_*CH_2_O), 3.52 (s, 6H, OCH_3_), 3.29 (d, *J* = 13.4 Hz, 4H, ArCH*H_eq_*Ar), 2.03 (ses, *J* = 7.8 Hz, 4H, OCH_2_*CH_2_*CH_3_), 1.99 (ses, *J* = 7.3 Hz, 4H, OCH_2_*CH_2_*CH_3_), 1.10 (t, *J* = 7.4 Hz, 6H, OCH_2_CH_2_*CH_3_*), 1.04 (t, *J* = 7.4 Hz, 6H, OCH_2_CH_2_*CH_3_*). ^13^C NMR (CDCl_3_, 100 MHz) δ (ppm): 158.84, 157.00, 156.87, 135.38, 135.00, 133.20, 131.48, 131.47, 131.10, 130.53, 129.90, 129.22, 128.93, 128.79, 127.34, 127.00, 125.87, 125.32, 124.37, 124.22, 123.52, 123.29, 122.29, 118.73, 118.09, 117.13, 116.12, 114.70, 96.17, 95.14, 87.71, 87.04, 77.23, 76.79, 71.00, 67.36, 59.28, 30.97, 23.40, 23.21, 10.54, 10.22. ESI-MS m/z calcd for C_96_H_80_O_8_: [M+H]^+^ 1391.6, found 1390.9.

**Scheme S5.** Synthesis of calixarene **CmPy**.

**Compound CmPy.** A 25 mL Schlenk tube was charged with compound **12** (77 mg, 0.12 mmol), compound **9** (60 mg, 0.14 mmol), Pd(PPh_3_)_2_Cl_2_ (4.4 mg, 0.0062 mmol), and CuI (1.7 mg, 0.0087). Three vacuum/N_2_ cycles were performed. Under a N_2_ atmosphere, dry THF (3 mL) and dry triethylamine (1 mL) were added. The resulting mixture was stirred at 65 °C for 36 hours. The mixture was diluted with CH_2_Cl_2_ (50 mL) and washed with water (3 x 50 mL). The organic phase was separated and dried over Na_2_SO_4_, and the solvent was removed under reduced pressure. **CmPy** was purified by column chromatography (silica gel, DCM) and obtained as a yellow powder (32 mg, 0.032 mmol) in 26% yield. ^1^H NMR (CDCl_3_, 400 MHz) δ (ppm): 8.69 (d, *J* = 9.1 Hz, 1H, H_py_), 8.64 (d, *J* = 9.1 Hz, 1H, H_py_), 8.26 – 8.13 (m, 6H, H_py_), 7.69 (d, *J* = 8.8 Hz, 2H, H_ph_), 7.06 (s, 2H, ArH), 7.01 (d, *J* = 8.8 Hz, 2H, H_ph_), 6.75 – 6.59 (m, 9H, ArH), 4.52 (d, *J* = 13.4 Hz, 2H, ArCH*H_ax_*Ar), 4.50 (d, *J* = 13.4 Hz, 2H, ArCH*H_ax_*Ar) 4.19 (t, *J* = 4.5 Hz, 2H, O*CH_2_*CH_2_OH), 4.04 (m, 2H, O*CH_2_*CH_2_OH), 4.01 – 3.82 (m, 8H, O*CH_2_*CH_2_CH_3_), 3.25 (d, *J* = 13.4 Hz, 2H, ArCH*H_eq_*Ar), 3.21 (d, *J* = 13.4 Hz, 2H, ArCH*H_eq_*Ar), 2.04 – 1.89 (m, 8H, OCH_2_*CH_2_*CH_3_), 1.13 – 0.95 (m, 12H, OCH_2_CH_2_*CH_3_*). ^13^C NMR (CDCl_3_, 100 MHz) δ (ppm): 158.9, 157.7, 156.8, 156.5, 136.0, 135.4, 135.1, 134.4, 133.3, 131.9, 131.8, 131.1, 130.8, 129.8, 129.7, 128.4, 128.3, 128.2, 128.1, 127.9, 126.4, 126.1, 125.1, 125.0, 124.3, 124.3, 122.1, 119.2, 118.6, 116.5, 116.1, 114.8, 96.7, 95.4, 87.6, 86.7, 77.3, 76.8, 76.6, 69.3, 61.4, 31.1, 31.0, 23.3, 23.3, 23.3, 10.4, 10.3, 10.3. ESI-MS m/z calcd for C_68_H_64_O_6_: [M+H]^+^ 977.9, found 977.5. ε_max_: 67150 M^-1^cm^-1^. Φ(380 nm): 75%.

# **Polymer** synthesis

**PU-CPy.** An oven-dried 100 mL Schlenk flask equipped with a magnetic stirring bar was charged with pTHF (*M_n_*= 2000 g/mol, 3.1 g, 1.5 mmol) and **CPy** (1.0 mg, 0.0007 mmol). Three vacuum/Ar cycles were performed. Under an Ar atmosphere, HMDI (1.2 mL, 5.0 mmol), dry THF (15 mL), and two drops of dibutyltin dilaurate were added. The reaction mixture was stirred at 45 °C for 3 hours. A solution of 1,4-butanediol (0.29 mL, 3.3 mmol) in 10 mL of dry THF was added. After 24 hours of stirring at 45 °C, the reaction was quenched by the addition of 20 mL of methanol. The mixture was precipitated in cold hexane (600 mL). The precipitate was collected by filtration and dried in vacuo for 48 hours at 60 °C. **PU-CPy** was obtained as a pale-yellow rubbery solid (4.5 g) in 98% yield. **CPy** concentration in **PU-CPy**: 0.023 wt%, 2.07 mol%. Number of **CPy** per polymer chain: 0.022. ^1^H NMR (CDCl_3_, 400 MHz) δ (ppm): 4.83 (bs, 1H), 4.54 (bs, 1H), 4.09 (bs, 3H), 3.78 (bs, 1H), 3.44 (bs, 23H), 2.01 (d, *J* = 11.6 Hz, 1H), 1.82 – 0.91 (m, 43H). SEC (THF): M*_n_* = 133 kDa, M*_w_* = 250 kDa, Đ = 1.9.

**Scheme S6.** Synthesis of **PU-CmPy**.

**PU-CmPy.** An oven-dried 100 mL Schlenk flask equipped with a magnetic stirring bar was charged with pTHF (*M_n_*= 2000 g/mol, 3.1 g, 1.5 mmol) and compound **CmPy** (1.5 mg, 0.0015 mmol). Three vacuum/Ar cycles were performed. Under an Ar atmosphere, HMDI (1.2 mL, 5.0 mmol), dry THF (15 mL), and two drops of dibutyltin dilaurate were added. The reaction mixture was stirred at 45 °C for 3 hours. A solution of 1,4-butanediol (0.29 mL, 3.3 mmol) in 10 mL of dry THF was added. After 48 hours of stirring at 45 °C, the reaction was quenched by the addition of 10 mL of methanol. The mixture was precipitated in cold hexane (600 mL). The precipitate was collected by filtration and dried in vacuo for 48 hours at 60 °C. **PU-CmPy** was obtained as a pale-yellow rubbery solid (4.6 g) in 99% yield. **CmPy** concentration in **PU-CmPy**: 0.032 wt%, 5.74 mol%. Number of **CmPy** per polymer chain: 0.056. ^1^H NMR (CDCl_3_, 400 MHz) δ (ppm): 4.83 (bs 1H), 4.55 (bs, 1H), 4.08 (bs, 3H), 3.77 (s, 1H), 3.43 (bs, 27H), 2.00 (d, *J* = 11.7 Hz, 2H), 1.87 – 0.90 (m, 49H). SEC (THF): M*_n_* = 173 kDa, M*_w_* = 296 kDa, Đ = 1.7.

**Scheme S7.** Synthesis of **PU**.

**PU.** An oven-dried 100 mL Schlenk flask equipped with a magnetic stirring bar was charged with pTHF (*M_n_*= 2000 g/mol, 3.0 g, 1.5 mmol). Three vacuum/Ar cycles were performed. Under an Ar atmosphere, HMDI (1.2 mL, 5.0 mmol), dry THF (15 mL), and two drops of dibutyltin dilaurate were added. The reaction mixture was stirred at 45 °C for 3 hours. A solution of 1,4-butanediol (0.29 mL, 3.3 mmol) in 10 mL of dry THF was added. After 24 hours of stirring at 45 °C, the reaction was quenched by the addition of 5 mL of methanol. The mixture was precipitated in cold hexane (600 mL). The white precipitate was collected by filtration and dried in vacuo for 48 hours at 60 °C. **PU** was obtained as a white solid (4.4 g) in 90% yield. ^1^H NMR (CDCl_3_, 400 MHz) δ (ppm): 4.82 (bs, 1H), 4.54 (bs, 1H), 4.09 (bs, 3H), 3.79 (bs, 1H), 3.43 (bs, 19H), 2.01 (d, *J* = 11.8 Hz, 1H), 1.88 – 0.90 (m, 38H). SEC (THF): M*_n_* = 58 kDa, M*_w_* = 105 kDa, Đ = 1.8.

**0.025CPyMeinPU, 0.05CPyMeinPU, 0.1CPyMeinPU.** In a 25 mL round-bottom flask, **PU** (300 mg) was dissolved in 10 mL of THF. **CPyMe** (0.075 mg, 0.15 mg, or 0.30 mg, respectively) was added, and the mixture was stirred at room temperature for 2 hours. The solution was transferred to a Teflon Petri dish, and the solvent was evaporated overnight under ambient conditions. The films were dried in vacuo for 48 h at 60 °C.

**Film preparation.** Uniform films were prepared by compression molding the synthesized polymers at 120 °C, using two poly(tetrafluoroethylene) (PTFE) sheets with PTFE spacers with a thickness of ca. 0.25 mm. The pressure was gradually increased over 5 cycles (0.80 tons increase per cycle), reaching a maximum of 4 tons, with each cycle lasting approximately 20 seconds. The films were subjected to the maximum pressure and temperature for 3 minutes, and then slowly cooled to room temperature over the course of a two-hour period while being held between the metal plates. The method produced films with a thickness of 0.22–0.23 mm.

# Thermal properties

**Figure S1.** Differential scanning calorimetry traces of **PU-CPy**. The measurements were conducted under N_2_. The heating and cooling rates were 10 °C/min.

**Figure S2.** Differential scanning calorimetry traces of **PU-CmPy**. The measurements were conducted under N_2_. The heating and cooling rates were 10 °C/min.

**Figure S3.** Differential scanning calorimetry traces of **PU**. The measurements were conducted under N_2_. The heating and cooling rates were 10 °C/min.

**Figure S4.** Thermogravimetric analysis trace of **PU-CPy**. The TGA experiments were conducted under N_2_ at a heating rate of 10 °C/min.

**Figure S5.** Thermogravimetric analysis trace of **PU-CmPy**. The TGA experiments were conducted under N_2_ at a heating rate of 10 °C/min.

**Figure S6.** Thermogravimetric analysis trace of **PU**. The TGA experiments were conducted under N_2_ at a heating rate of 10 °C/min.

# Mechanical properties

**Figure S7.** Stress-strain curves of **PU-CPy**. Data were obtained from six different specimens. The experiments were conducted at a strain rate of 2.5% strain per second at room temperature. The material was deformed from 0% strain to 480% strain.

**Figure S8.** Stress-strain curves of **PU-CmPy**. Data were obtained from four different specimens. The experiments were conducted at a strain rate of 2.5% strain per second at room temperature. The material was deformed from 0% strain to 480% strain.

**Figure S9.** Stress-strain curves of **PU**. Data were obtained from three different specimens. The experiments were conducted at a strain rate of 2.5% strain per second at room temperature. The material was deformed from 0% strain to 480% strain.

# Solid-state crystal structure of CPy


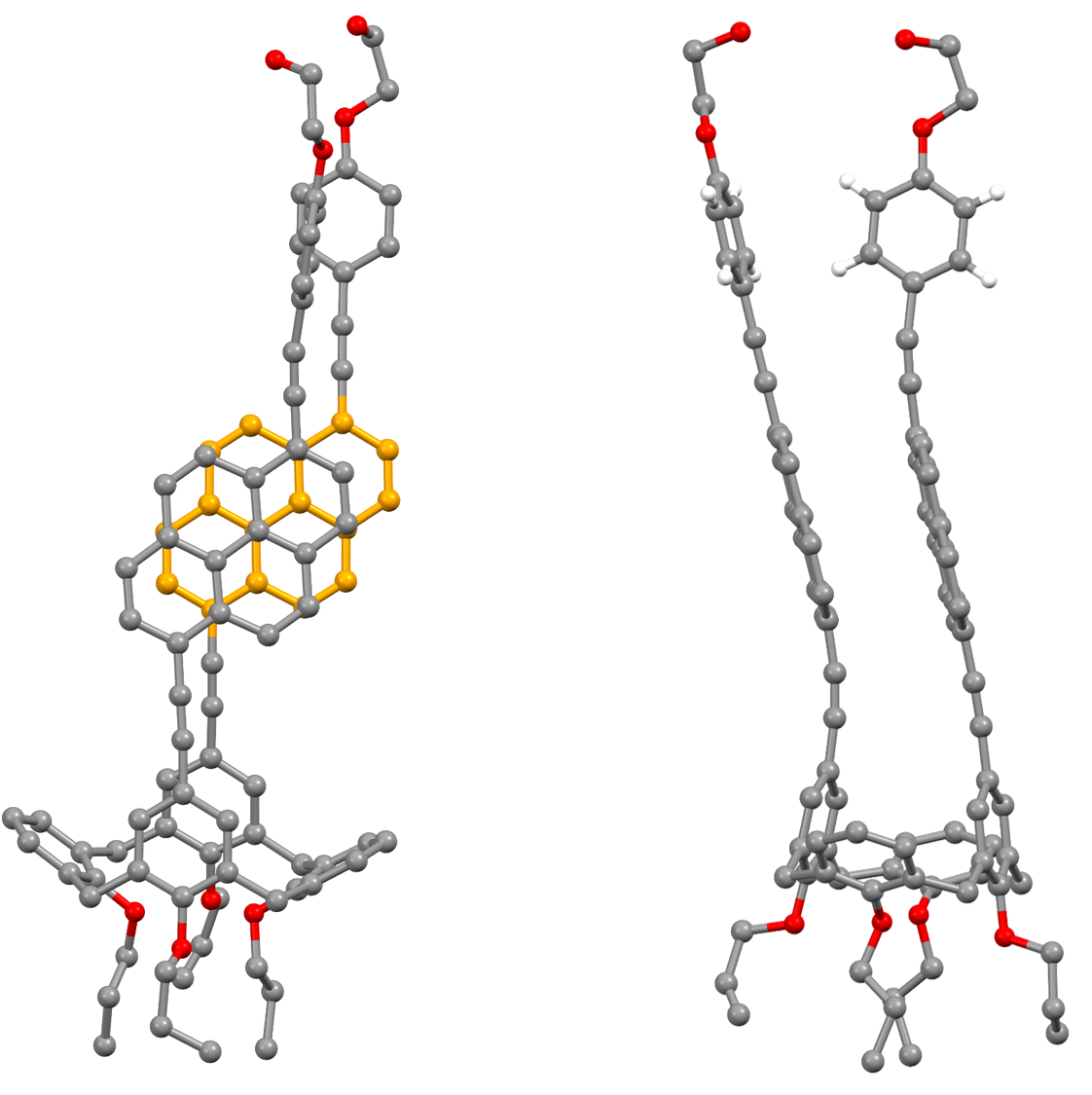


**Figure S10.** Views of the crystal structure of **CPy** that display (left) the stacked arrangement of the pyrene rings and (right) the T-shaped π-π interaction between the terminal benzene rings. Most of the hydrogen atoms and solvent molecules have been omitted for clarity.

# ^1^H NMR spectra of CPy, CmPy, and CPyMe

**Figure S11.** Comparison between the ^1^H NMR spectra of **CmPy** (trace a), **CPy** (trace b), and **CPyMe** (trace c). The spectra were recorded in CDCl_3_ at 400 MHz and 25 ºC.

# DOSY spectrum of CPy

**Figure S12.** DOSY spectrum (Bayesian mode) of a 5 mM solution of **CPy** in CDCl_3_ at 400 MHz and 25 ºC. A single diffusion coefficient of 4.0 x 10^-6^ cm^2^ s^-1^ is observed, indicating the presence of a single species, and thereby ruling out intermolecular aggregation.

**Figure S13.** DOSY spectrum (Bayesian mode) of a 5 mM solution of **CmPy** in CDCl_3_ at 400 MHz and 25 ºC.

# Emission spectrum of CPyMe

**Figure S14.** Comparison between the emission profiles of 10^-6^ M dichloromethane solutions of **CPy** and **CPyMe** upon irradiation with 380 nm light.

# Concentration dependency of luminescence spectra of CPy and CmPy

**Figure S15.** Emission spectra of dichloromethane solutions of (a) **CPy** and (b) **CmPy** at concentrations ranging from 10^-6^ to 8 x 10^-6^ M. All spectra were recorded at room temperature using an excitation wavelength of 380 nm.

# Emission decays of CPy and CPyMe

**Figure S16.** Decay profiles of the emission of (a) 10^-6^ M **CPy** in DCM (monitored at 600 nm) and (b) 10^-6^ M **CmPy** in DCM (monitored at 440 nm) using an excitation wavelength of 405 nm.

# Photophysical properties of PU-CPy in solution

**Figure S17.** (a) Absorption and (b) photoluminescence spectra of **PU-CPy** and **CPy** in dichloromethane. (c) excitation spectra of **PU-CPy** at different emission wavelengths (415, 440, 470, 600 nm). The concentration of luminophores was 1.0 x 10^-6^ M in all the measurements. The luminescence spectra were recorded using an excitation wavelength of 380 nm. (d) Comparison between the excitation profile of **PU-CPy** recorded at 415 nm and the absorption profile of **CPy**. This comparison is presented because the emission band at 415 nm of **PU-CPy** (black line in panel b) is absent in **CPy** (red line). The excitation profile at this emission wavelength (415 nm) does not match the absorption profile of **CPy**. Thus, this band was attributed to a fluorescent impurity likely formed during polymer synthesis.

# Stress-strain curves for two stress and release cycles

**Figure S18.** Stress−strain curves recorded in the (a) first and (b) second stretch and release cycles of a **PU-CPy** film (brown trace) and correlation with the monomer-to-excimer ratio (blue trace) recorded upon stretching and releasing.

# Fluorescence intensity ratio during stress relaxation

**Figure S19.** Stress relaxation curve (brown trace) and I_470_/I_580_ ratio (blue trace) of a **PU-CPy** film recorded for 300 seconds after fast stretching (30%/s strain rate).

# Fluorescence intensity ratio during one-hour continuous irradiation


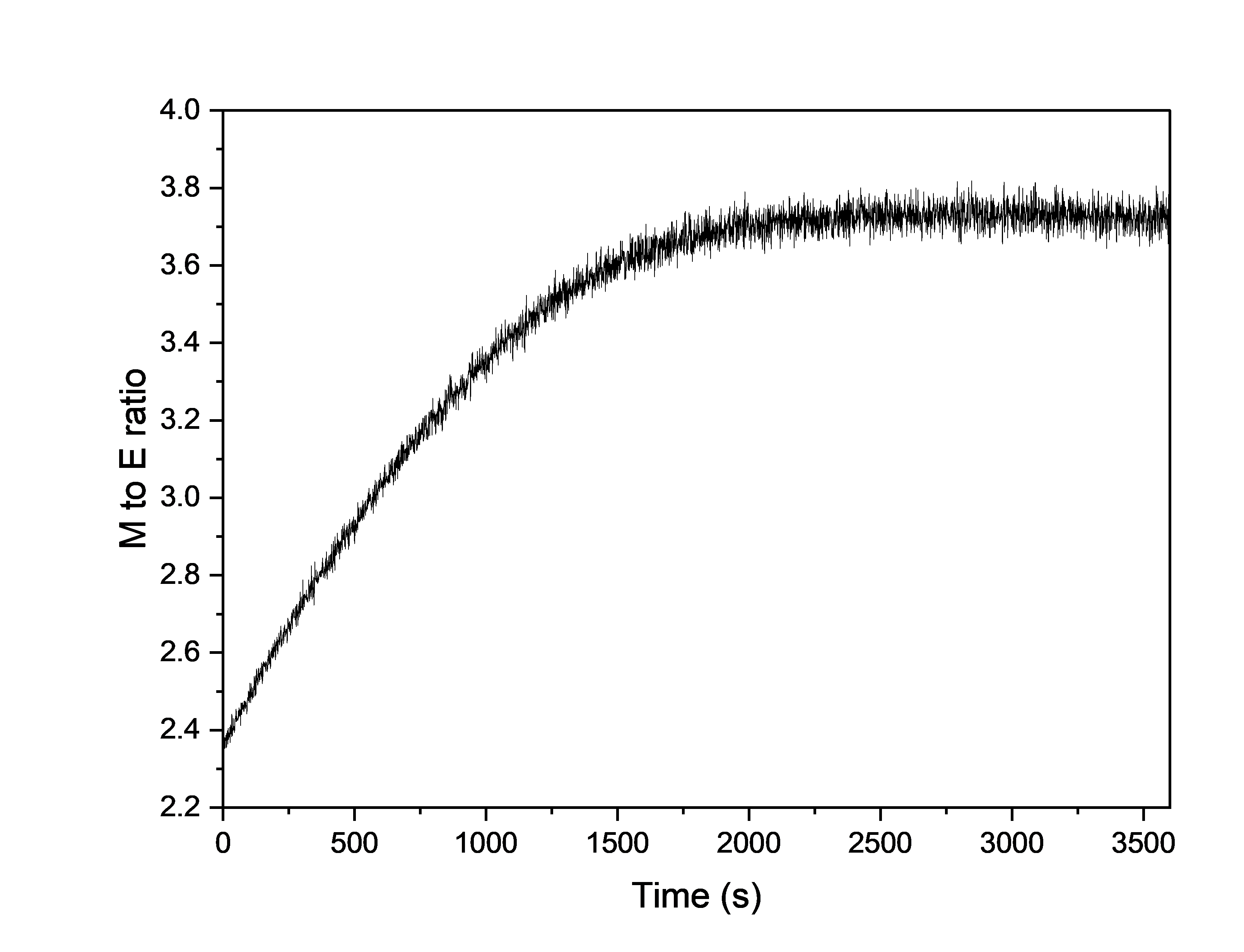

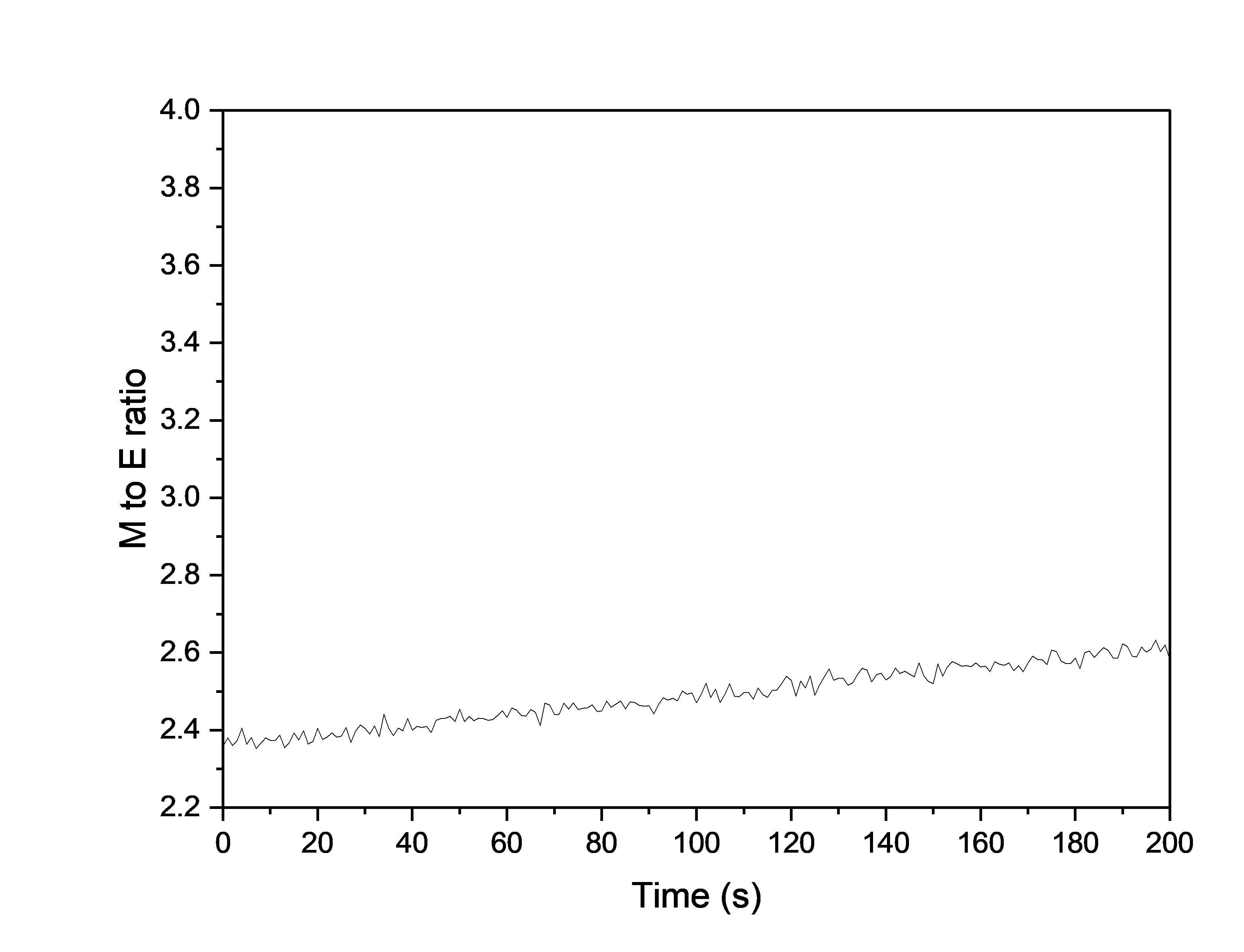

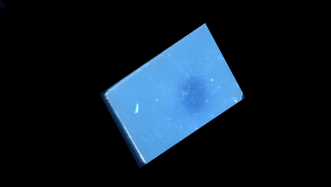


**Figure S20.** Variation of 𝐼_470_/𝐼_580_ ratio of **PU-CPy** upon continuous irradiation with a 365 nm LED and photography of the polymer film after one hour of irradiation.

# Stress and fluorescence response at different strain rates

**Figure S21.** Stress-strain curves (brown traces) and monomer to excimer ratio (blue dots) of **PU-CPy** upon uniaxial tensile deformation at strain rates ranging from 1%/s to 30% s^-1^.

# Luminescence properties of PU-CmPy

**Figure S22.** Photoluminescence spectra of **PU-CmPy** and **CmPy** in dichloromethane. The concentrations of luminophores were 1.0 x 10^-6^ M. The luminescence spectra were recorded using an excitation wavelength of 380 nm. Photoluminescence spectrum of **PU-CPy** film recorded using an excitation wavelength of 365 nm.

#

# Luminescence of PU-CmPy upon elongation


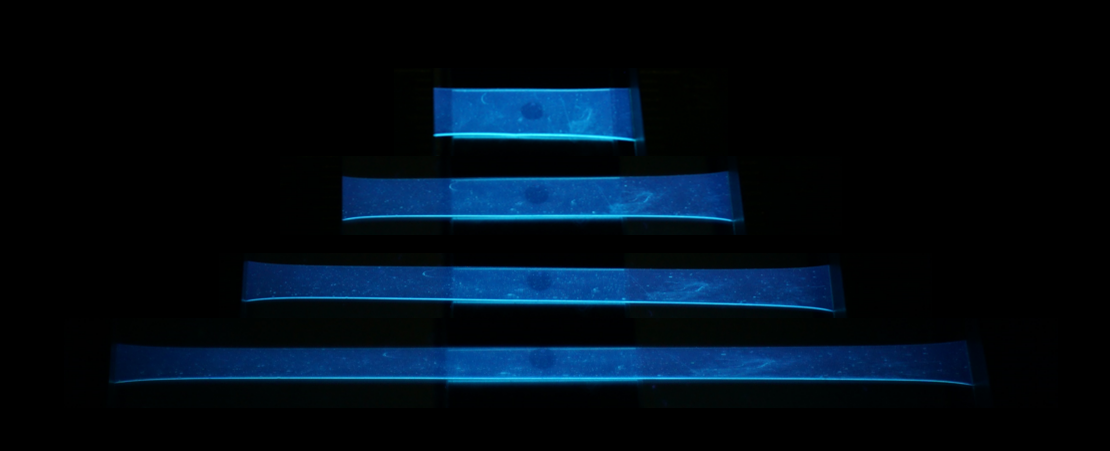


**Figure S23.** Photographs of **PU-CmPy** film at 0%, 100%, 200%, and 300% strain (from top to bottom) under 365 nm light irradiation.

# Luminescence properties and correlation between stress-strain curves and the fluorescence intensity ratio of CPyMe blends in PU upon stretching

**Figure S24.** (a) Emission spectra of **0.025CPyMeinPU**, **0.05CPyMeinPU**, and **0.1CPyMeinPU** films upon uniaxial tensile deformation from 0% (yellow profiles) to 480% (blue profiles) strain at a strain rate of 2.5% and under irradiation with 365 nm light, and (b) correlation between stress-strain curves and I_470_/I_580_ ratio.

# Luminescence of 0.025CPyMeinPU upon elongation


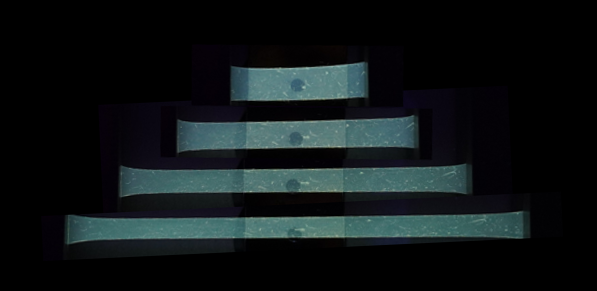


**Figure S25.** Photographs of **0.025CPyMeinPU** film at 0%, 100%, 200% and 300% strain (from top to bottom) under 365 nm light irradiation.

# NMR spectra

**Figure S26.** ^1^H NMR spectrum of **1a**.

**Figure S27.** ^1^H NMR spectrum of **2**.

**Figure S28.** ^1^H NMR spectrum of **2a**.

**Figure S29.** ^1^H NMR spectrum of **3**.

**Figure S30.** ^1^H NMR spectrum of **5**.

**Figure S31.** ^1^H NMR spectrum of **6**.

**Figure S32.** ^1^H NMR spectrum of **7**.

**Figure S33.** ^1^H NMR spectrum of **8**.

**Figure S34.** ^1^H NMR spectrum of **4**.

**Figure S35.** ^1^H NMR spectrum of **10**.

**Figure S36.** ^1^H NMR spectrum of **11**.

**Figure S37.** ^1^H NMR spectrum of **12**.

**Figure S38.** ^1^H NMR spectrum of **CPy**.

**Figure S39.** ^13^C NMR spectrum of **CPy**.

**

**Figure S40.** ^1^H NMR spectrum of **CmPy**.

**Figure S41.** ^13^C NMR spectrum of **CmPy**.

**Figure S42.** ^1^H NMR spectrum of **CPyMe**.

**Figure S43.** ^13^C NMR spectrum of **CPyMe**.

**Figure S44.** ^1^H NMR spectrum of **PU-CPy**.

**Figure S45.** ^1^H NMR spectrum of **PU-CmPy**.

**Figure S46.** ^1^H NMR spectrum of **PU**.

**Single crystal X-ray Diffraction**

Single crystal data were collected at 200 K with a Bruker D8 Venture diffractometer equipped with a Photon II detector, using a microfocus radiation source (Cu Kα: λ = 1.54184 Å). The intensity data were integrated from several series of exposure frames covering the sphere of reciprocal space. Data reductions were performed with APEX5. Absorption corrections were applied using the program CrysAlisPro.^[41]^ The structures were solved by intrinsic phasing with the program SHELXT.^[42]^ Fourier analysis and refinement were performed by the full–matrix least–squares methods based on F^2^ using SHELXL–2017 implemented in Olex2 software (version 1.5).^[43]^ Non–H atoms were refined anisotropically. One of the n-propyl groups was found disordered in two positions, which were refined with site occupancy factors of 0.5 each. One of the two phenyl alkyne residues was found disordered in two positions, which were refined with site occupancy factors of 0.5 each. Two dichloromethane molecules of crystallization were identified. One was refined with site occupancy factors of 1, whereas the second molecule was located close to the disordered phenyl alkyne residue and it was refined with a site occupancy factor of 0.25.

**Table S1** Crystal data and structure refinement for **CPy**

| Empirical formula | C_97_H_82_Cl_2.5_O_8_ |
| --- | --- |
| Formula weight | 1464.25 |
| Temperature/K | 200.15 |
| Crystal system | triclinic |
| Space group | P-1 |
| a/Å | 12.6862(8) |
| b/Å | 17.0210(7) |
| c/Å | 19.7951(13) |
| α/° | 90.164(4) |
| β/° | 104.081(6) |
| γ/° | 92.876(4) |
| Volume/Å^3^ | 4140.2(4) |
| Z | 2 |
| ρ_calc_g/cm^3^ | 1.175 |
| μ/mm^‑1^ | 1.295 |
| F(000) | 1541.0 |
| Crystal size/mm^3^ | 0.2 × 0.17 × 0.02 |
| Radiation | CuKα (λ = 1.54184) |
| 2Θ range for data collection/° | 4.602 to 133.196 |
| Index ranges | -15 ≤ h ≤ 13, -20 ≤ k ≤ 20, -23 ≤ l ≤ 23 |
| Reflections collected | 82519 |
| Independent reflections | 14535 [R_int_ = 0.1398, R_sigma_ = 0.0949] |
| Data/restraints/parameters | 14535/216/1081 |
| Goodness-of-fit on F^2^ | 1.020 |
| Final R indexes [I>=2σ (I)] | R_1_ = 0.0966, wR_2_ = 0.2694 |
| Final R indexes [all data] | R_1_ = 0.1690, wR_2_ = 0.3421 |
| Largest diff. peak/hole / e Å^-3^ | 0.94/-0.32 |
